# Evaluation of Selected Laboratory Parameters as Predictive Biomarkers of Clinical Response to Dupilumab Therapy in Patients with Atopic Dermatitis

**DOI:** 10.3390/biomedicines14081767

**Published:** 2026-08-06

**Authors:** Katarzyna Waligóra-Dziwak, Piotr Jerzy Skrzypczak, Dorota Jenerowicz

**Affiliations:** 1 Department of Dermatology, Poznan University of Medical Sciences, 60-355 Poznan, Poland; 2 Data Science in Medicine, Department of Computer Science and Statistics, Poznan University of Medical Sciences, ul. Rokietnicka, 60-806 Poznan, Poland; 3 Department of Thoracic Surgery, Poznan University of Medical Sciences, 60-569 Poznan, Poland

**Keywords:** atopic dermatitis, biomarkers, dupilumab

## Abstract

**Background:** Clinical response to dupilumab in patients with atopic dermatitis is variable, and no established laboratory biomarkers currently enable reliable identification of patients likely to achieve a predictable response prior to treatment initiation or early during therapy. **Methods:** This prospective, real-world study conducted in a Polish population evaluated the discriminative capacity of selected laboratory biomarkers for predicting short- and long-term clinical response to dupilumab in patients with atopic dermatitis. **Results:** Lactate dehydrogenase (LDH) was the most consistently informative continuous baseline biomarker (area under the receiver operating characteristic curve [AUC] for at least 75% improvement in the Eczema Area and Severity Index [EASI-75] at week 16: 0.60; EASI-90 at week 16: 0.63; EASI-75 at week 40: 0.66; EASI < 7 at week 40: 0.64). The systemic inflammatory response index (SIRI) was the second most stable baseline predictor (AUC = 0.63 and 0.64 for EASI-75 and EASI < 7 at week 40, respectively) and demonstrated the highest overall discriminative performance among week 16 biomarkers. **Conclusions:** LDH and SIRI appear to be the most promising candidates for further evaluation in larger cohorts owing to their cross-outcome and cross-time consistency rather than a single dominant AUC value. The predictive potential of individual biomarkers, while detectable, remained below the threshold of clinical utility for every biomarker–outcome combination examined. Composite multi-biomarker models may be required to achieve clinically meaningful predictive accuracy.

## 1. Introduction

Atopic dermatitis (AD) represents a chronic, recurrent inflammatory skin disease, marked by cutaneous dryness, erythematous lesions, and severe pruritus [1]. Its phenotypic expression is heterogeneous and strongly influenced by age [2]. As one of the most widespread dermatologic conditions worldwide, AD constitutes a considerable clinical and socioeconomic burden for affected individuals [3]. Accordingly, individual therapeutic approaches are crucial for optimizing clinical outcomes and alleviating this burden.

Dupilumab is a fully human IgG4 monoclonal antibody that acts as an inhibitor of the interleukin-4 (IL-4) and interleukin-13 (IL-13) signaling pathways—cytokines that play a significant role in the pathophysiology of AD [4]. Dupilumab inhibits IL-4 signaling through the type I receptor (IL-4Rα/γc) and also blocks signaling mediated by both IL-4 and IL-13 through the type II receptor (IL-4Rα/IL-13Rα) [5,6].

Clinical response to dupilumab therapy in patients with severe AD is variable. AD is a highly heterogeneous disease, and currently there are no established laboratory markers capable of identifying patient subgroups with a predictable clinical response prior to treatment initiation or early in the course of treatment [7]. A better understanding of the relationship between laboratory parameters and the clinical efficacy of dupilumab in the treatment of AD might enable greater personalization of the therapeutic approach.

In clinical studies of AD, treatment with dupilumab has been associated with reductions in type 2 immune biomarkers, such as total IgE and allergen-specific IgE in serum [8]. During dupilumab therapy, decreases in lactate dehydrogenase (LDH) activity—a marker of disease activity and severity—have been observed in both adults and adolescents with AD [9,10]. Serum eosinophilic cationic protein (ECP) has been reported in the literature as a marker reflecting disease severity in AD [11,12]. Eosinophilia has been noted as an adverse effect of dupilumab treatment, yet it is also described as a parameter correlating with disease severity [13]. *Malassezia*-specific serum IgE has been proposed as a potential outcome biomarker in patients with AD undergoing dupilumab therapy, with current evidence primarily focusing on its association with the development of dupilumab-associated head and neck dermatitis [14]. The impact of dupilumab on the aforementioned laboratory parameters has been described in the literature; however, the correlation between treatment efficacy—particularly long-term—and the effects of dupilumab on selected laboratory parameters remains under investigation, with some studies reporting partially conflicting results [7,15,16].

The present study was designed to evaluate the discriminative capacity of selected laboratory biomarkers for predicting short- and long-term clinical response to dupilumab in patients with severe AD. To the best of the authors’ knowledge, this is the first real-world study to assess the predictive value of ECP alongside inflammatory indices, including the systemic inflammatory response index (SIRI) and eosinophil-to-lymphocyte ratio (ELR), with complete skin clearance incorporated as one of the predefined efficacy endpoints. Furthermore, it represents the first investigation of this kind conducted in a Polish population.

## 2. Materials and Methods

### 2.1. Study Design

The prospective single-centered real-world study was conducted in Polish patients from February 2024 to February 2026. Additionally, routine laboratory results obtained prior to study initiation were included in the analysis with written informed consent from all participants. A total of 40 patients with a confirmed diagnosis of severe AD were enrolled in the study. All patients underwent clinical examination, and disease severity was evaluated using the Eczema Area and Severity Index (EASI) at baseline, at week 16, and at week 40 in those eligible for long-term treatment.

Blood samples for laboratory analyses were collected prior to initiation of dupilumab therapy (baseline) and at week 16 of treatment (±4 weeks). Laboratory assessments included determination of (1) hematological parameters of the white blood cell differential (total WBC count, absolute lymphocyte, neutrophil, monocyte, and eosinophil counts, eosinophil percentage [Sysmex XN-1000, Kobe, Japan]); (2) composite inflammatory and immune ratios (eosinophil-to-lymphocyte ratio [ELR], systemic inflammatory response index [SIRI], computed as [neutrophils × monocytes]/lymphocytes); (3) ECP (UniCAP 100, CAP^TM^ System, Pharmacia & Upjohn Diagnostics AB, Uppsala, Sweden); (4) LDH activity (Cobas 8000, c502, Roche, Mannheim, Germany); and (5) immunoglobulin E parameters (total serum IgE, *Malassezia*-specific IgE [UniCAP 100, CAP^TM^ System, Pharmacia & Upjohn Diagnostics AB, Uppsala, Sweden]).

The study objective was to determine whether baseline laboratory parameters could serve as reliable predictors of treatment response, defined as the attainment of EASI-75, EASI-90, and EASI-100 (at least 75% or 90% or 100% improvement in the Eczema Area and Severity Index, respectively) thresholds at week 16 and week 40 of therapy. The achievement of an EASI score below 7, corresponding to low disease activity, was additionally assessed as a clinically relevant absolute threshold. In addition, laboratory parameters measured at week 16 were evaluated for their predictive value with respect to treatment outcomes at week 40. The dual time-point evaluation at weeks 16 and 40 was designed to capture both short-term induction efficacy and sustained long-term therapeutic benefit. All analyses are presented as exploratory.

The study was approved by Institutional Research Ethics Committee and conducted in accordance with the Declaration of Helsinki and applicable regulations.

### 2.2. Patients

The study group consisted of 40 patients aged 19–65 years, including both male and female participants, diagnosed with moderate-to-severe AD. Participants were eligible for inclusion if they were qualified for a dupilumab treatment under the Polish National Health Fund Program for severe AD, were in good general health at the time of blood sampling, had no history of malignancy, liver disease, or active parasitic infections, were not pregnant, and provided written informed consent. Exclusion from the study or discontinuation of participation occurred in the event of pregnancy; a history of malignancy within the preceding 5 years (excluding non-invasive basal cell carcinoma and squamous cell carcinoma); significant hepatic dysfunction; active parasitic infections; contraindications to dupilumab therapy; poor general health at the time of blood sampling; use of systemic glucocorticosteroids at the time of blood collection; or withdrawal of informed consent.

### 2.3. Treatment Dosage and Concomitant Medications

Dupilumab was administered subcutaneously at a dose of 300 mg every two weeks, following a 600 mg loading dose. The treatment duration was 16 weeks, with possible extension to at least 40 weeks for eligible patients (who did not meet exclusion or discontinuation criteria). At the time of blood sampling, none of the patients included in the analysis were treated with systemic glucocorticosteroids. The use of concomitant topical therapies was allowed throughout the treatment period at the treating physician’s discretion.

### 2.4. Statistical Analysis

All continuous variables were assessed for distributional normality using the Shapiro–Wilk test. Continuous variables were reported as medians with interquartile ranges (IQR). Confidence intervals for medians were computed by the distribution-free order-statistic (binomial) method and corroborated by bootstrap resampling; the Hodges–Lehmann estimator was used to estimate the median paired difference, and corresponding 95% confidence intervals were calculated using the Wilcoxon signed-rank procedure. Categorical variables were expressed as absolute frequencies and percentages with 95% Wilson score confidence intervals. Ordinal variables were summarized using the full frequency distribution.

The longitudinal evolution of continuous laboratory biomarkers from baseline to week 16 was evaluated using the Wilcoxon signed-rank test for paired samples. Total serum IgE exceeded the upper assay quantification limit (5000 kU/L) in 16 of 40 patients (40.0%); to avoid substitution-dependent inference, the ordinal IgE class was the pre-specified representation for the logistic-regression analyses. The rank-based analyses (Wilcoxon signed-rank test and the area under the ROC curve) are invariant to the ceiling substitution, because every censored value exceeds the maximum uncensored value (4173 kU/L); the continuous form was therefore used only for those rank-based displays and as a labeled sensitivity analysis (Supplementary Material S1). For ordinal IgE class distributions, the Stuart–Maxwell test of marginal homogeneity was applied. Paired proportions were compared with the exact McNemar test. Changes in clinical outcome measures assessed at baseline, week 16 and week 40 were analyzed using the Friedman test.

Receiver Operating Characteristic (ROC) curve analysis constituted the principal method for evaluating the discriminative capacity of individual baseline laboratory parameters with respect to each binary clinical outcome. The optimal discriminative threshold for each candidate biomarker was determined using the Youden index. Discrimination was reported as the area under the ROC curve with its 95% confidence interval (DeLong method for continuous predictors; stratified bootstrap for the ordinal IgE-class predictors), and sensitivity, specificity, positive predictive value and negative predictive value at the Youden operating point were each reported with exact binomial (Clopper–Pearson) 95% confidence intervals; the Youden-derived cut-offs were optimized in-sample and are therefore exploratory. Univariate logistic regression models were constructed for candidate predictors identified as the baseline laboratory biomarkers with the most consistent discriminative performance based on the cross-cutting synthesis of ROC analysis results.

Patient-level transitions in response status between weeks 16 and 40 were visualized through alluvial (Sankey-type) flow diagrams. A two-sided *p* value < 0.05 was considered indicative of statistical significance throughout all analyses. Analyses were conducted using the R Statistical language (version 4.5.2; [17]) and IBM SPSS Statistics (version 29.0). Further details of the statistical analysis are provided in Supplementary Material S1 and Appendix A.

## 3. Results

### 3.1. Baseline Characteristics

Forty Polish patients with diagnosed moderate-to-severe AD were included in the study; all were of Caucasian ancestry (65.0% male, 35.0% female), aged 19–65 years (median 32.5 years; IQR 25.0–41.0). Thirty-nine patients (97.5%) completed the full 40-week observation period; one patient was lost to follow-up after the week 16 assessment, yielding an evaluable sample of *n* = 39 for week 40 outcomes. The median disease duration was 27.0 years (IQR: 19.6–38.3), reflecting a population with long-standing, chronic disease. The median baseline EASI score was 26.5 (IQR: 22.0–38.4), confirming moderate-to-severe disease in all enrolled patients. The majority of patients (33/40; 82.5%) had received at least one prior systemic immunosuppressive therapy, most commonly cyclosporine A. Seven patients (17.5%) were treatment-naïve to systemic therapy. Atopic comorbidities were highly prevalent (Table 1).

### 3.2. Longitudinal Evolution of Laboratory Parameters

The temporal dynamics of the examined laboratory biomarkers from baseline to week 16 of dupilumab therapy were evaluated. The complete results are presented in Table 2. The total white blood cell count (*p* = 0.727), absolute neutrophil count (*p* = 0.195), and absolute monocyte count (*p* = 0.503) remained stable, indicating no measurable global leukocyte effect within this timeframe. In contrast, the absolute lymphocyte count rose significantly from 1.58 to 1.75 × 10^3^/μL (*p* = 0.006). Both the absolute eosinophil count and the eosinophil percentage exhibited upward trends without reaching formal significance. The eosinophil-to-lymphocyte ratio remained unchanged (*p* = 0.476), as the concurrent rise in both eosinophils and lymphocytes neutralized any net directional shift. Conversely, the systemic inflammatory response index (SIRI) declined significantly from 1.31 to 1.16 (*p* = 0.012). Serum LDH activity exhibited the most robust decline among all variables, decreasing from 236.0 to 182.0 U/L (*p* < 0.001). Serum ECP increased numerically from 18.55 to 23.20 μg/L (*p* = 0.097). Total serum IgE declined from a median of 3439.5 to 1698.5 kU/L (*p* < 0.001). The ordinal IgE class distribution, however, did not shift significantly (*p* = 0.731), exposing the insensitivity of coarse categorical boundaries to within-class quantitative changes. *Malassezia*-specific IgE likewise declined significantly from 4.10 to 2.41 kUA/L (*p* < 0.001).

### 3.3. Longitudinal Trajectory of Disease Severity

**Figure 1:**
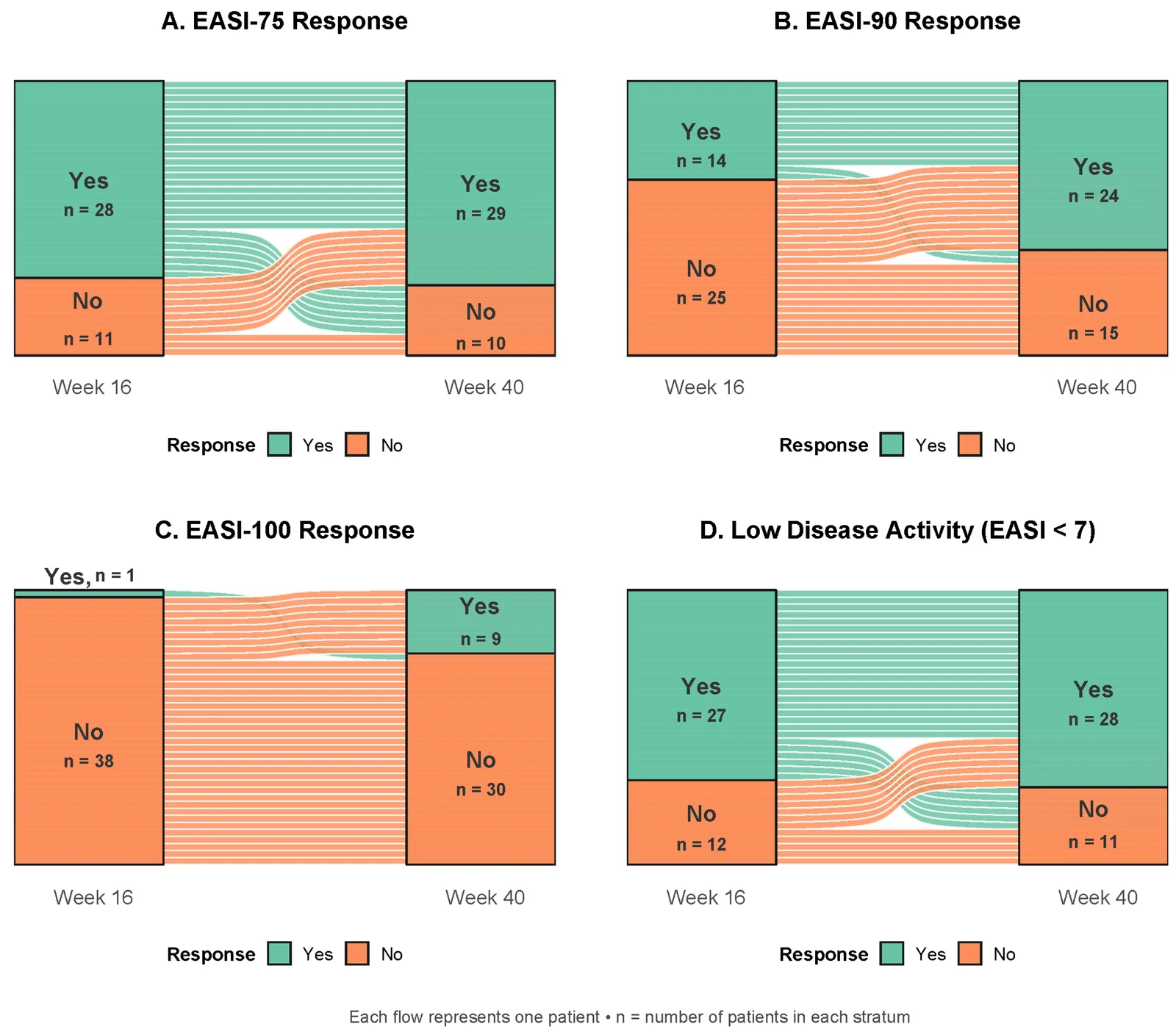
Patient-level transitions in EASI response thresholds between week 16 and week 40 of dupilumab therapy. Alluvial (Sankey) diagrams illustrate the flow of individual patients (*n* = 39 completed pairs) across responder categories for (**A**) EASI-75, (**B**) EASI-90, (**C**) EASI-100, and (**D**) low disease activity (EASI < 7). Each flow band represents a single patient. Green strata denote responders; orange strata denote non-responders.

Dupilumab therapy elicited a marked and sustained reduction in disease severity over the 40-week observation period, as quantified by the Eczema Area and Severity Index (Table 3). The analysis of categorical response endpoints (Table 4) revealed that the behavior of dupilumab’s therapeutic effect between weeks 16 and 40 is threshold-dependent. At the EASI-75 level, the proportion of responders was virtually identical at both time points. A similarly stable pattern was observed for the low disease activity target. A markedly different pattern emerged at the more stringent response thresholds. EASI-90 achievement rose from 35.0% at week 16 to 61.5% at week 40, representing a near-doubling of the responder proportion (*p* = 0.018). Even more striking was the trajectory of complete clearance: EASI-100 was attained by only 1/40 patient (2.5%) at week 16, yet by 9/39 patients (23.1%, 95% CI 12.0–40.0%) at week 40 (McNemar’s *p* = 0.030). Patient-level transitions in EASI response thresholds between week 16 and week 40 of dupilumab therapy are presented in Figure 1.

### 3.4. Predictive Performance of Laboratory Biomarkers (ROC Analysis)

#### 3.4.1. Baseline Predictors of Week 16 Response

Among week 16 outcomes, the most promising overall predictive landscape was observed for the low disease activity target (EASI < 7; Table 5). Several biomarkers attained AUC values (area under the receiver-operating characteristic curve) in the 0.61–0.67 range. Baseline total serum IgE (≤258 kU/L; AUC = 0.67; Youden J = 0.39; specificity 1.00, sensitivity 0.39), WBC count (≤6.89 × 10^3^/μL; AUC = 0.67; Youden J = 0.38; sensitivity 0.71, specificity 0.67), and monocyte count (≤0.64 × 10^3^/μL; AUC = 0.66; Youden J = 0.33) led this outcome. *Malassezia*-specific IgE (AUC = 0.63), ELR (AUC = 0.61), and ECP (AUC = 0.61) contributed additional, convergent signals; however, none of these biomarkers exceeded the AUC threshold of 0.70.

For the EASI-75 endpoint at week 16 (Table 5), no individual biomarker achieved an AUC that would show clinically useful discriminative performance. AUC values ranged from 0.50 (monocyte count, ECP) to 0.60 (LDH), with the vast majority clustering between 0.52 and 0.57, and the highest-performing candidate being baseline LDH (≥284 U/L; AUC = 0.60, Youden J = 0.27). The EASI-90 endpoint at week 16 (Table 5), yielded marginally higher AUC values. LDH emerged as the leading potential predictor (AUC = 0.63; cut-off ≤ 204 U/L; sensitivity 0.43, specificity 0.88, Youden J = 0.31), followed by ECP (AUC = 0.60; ≥28.90 μg/L; sensitivity 0.50, specificity 0.77). The remaining evaluated biomarkers demonstrated AUC values below 0.60 (Table 5 and Table 6).

#### 3.4.2. Baseline Predictors of Week 40 Response

For EASI-75 at week 40 (Table 7 and Table 8), LDH once again occupied the leading position among continuous biomarkers (AUC = 0.66; ≤238 U/L; Youden J = 0.38; sensitivity 0.68, specificity 0.70), followed by *Malassezia*-specific IgE (AUC = 0.64; ≤0.32 kUA/L; specificity 1.00, sensitivity 0.38) and SIRI (AUC = 0.63). For EASI-90 at week 40, the discriminative capacity of all biomarkers declined further: the highest AUC was 0.62 (lymphocyte count), with the remainder falling below 0.60. For EASI-100 at week 40—an outcome of particular clinical interest given the substantial late-responder phenomenon—continuous biomarkers performed poorly.

The low disease activity outcome at week 40 (Table 7 and Table 8) produced a predictive profile dominated by LDH (AUC = 0.64; ≤218 U/L; Youden J = 0.39; specificity 0.91, sensitivity 0.48), SIRI (AUC = 0.64; ≤1.28; Youden J = 0.33), and lymphocyte count (AUC = 0.62). The recurrence of LDH and SIRI at this threshold—positions them as the most consistent predictors within the continuous biomarker panel, even though their absolute discriminative capacity remained in the modest range.

**Figure 2:**
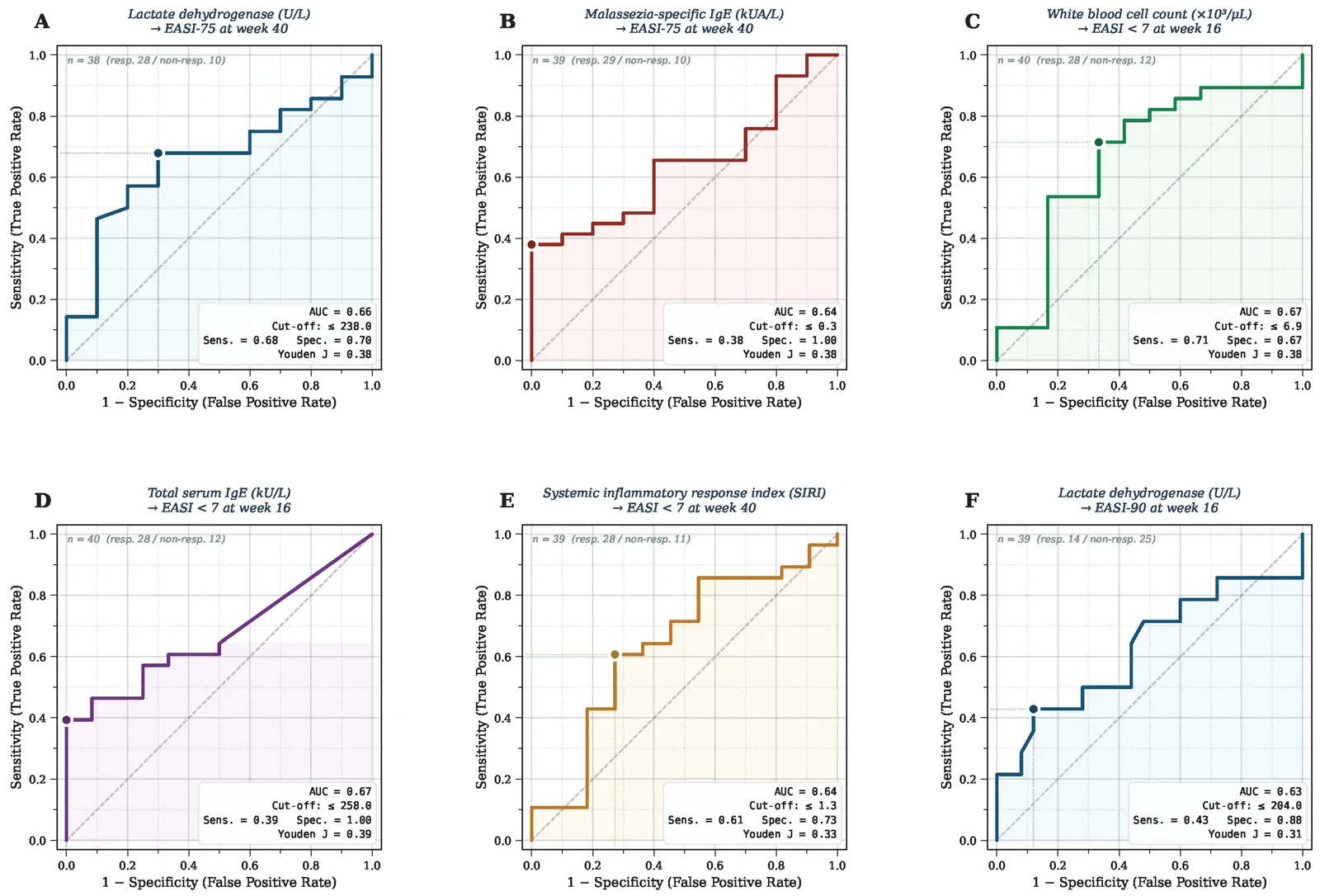
Receiver operating characteristic (ROC) curves for the six most discriminative baseline laboratory biomarker–outcome combinations identified in the cross-cutting synthesis of predictive performance. Panels depict: (**A**) lactate dehydrogenase → EASI-75 at week 40; (**B**) Malassezia-specific IgE → EASI-75 at week 40; (**C**) white blood cell count → low disease activity (EASI < 7) at week 16; (**D**) total serum IgE → low disease activity at week 16; (**E**) systemic inflammatory response index (SIRI) → low disease activity at week 40; (**F**) lactate dehydrogenase → EASI-90 at week 16. For each panel, the area under the curve (AUC), the optimal discriminative threshold determined by the Youden index (J = sensitivity + specificity − 1), and the corresponding sensitivity and specificity are annotated. The filled circle denotes the optimal operating point; dashed guide lines project its coordinates onto the axes. Sample sizes and responder/non-responder counts are indicated in each panel. All predictors are oriented such that lower baseline values favor a positive clinical outcome.

Receiver operating characteristic (ROC) curves for the six most discriminative baseline laboratory biomarker–outcome combinations identified in the cross-cutting synthesis of predictive performance are presented in Figure 2.

#### 3.4.3. 16-Week Predictors of Week 40 Response

Across all week 40 efficacy endpoints (EASI-75, EASI-90, EASI-100, and EASI < 7), SIRI demonstrated consistently the highest discriminative performance among the evaluated biomarkers (for EASI-75 outcome: AUC = 0.65; ≤1.18; Youden J = 0.36; sensitivity 0.66; specificity 0.70; for EASI-90 outcome: AUC = 0.64; ≤0.92; Youden J = 0.38; sensitivity 0.58; specificity 0.80; for EASI-100 outcome: AUC = 0.69; ≤0.75; Youden J = 0.39; sensitivity 0.56; specificity 0.83; for EASI < 7 outcome: AUC = 0.66; ≤1.18; Youden J = 0.41; sensitivity 0.68; specificity 0.73), with AUC values remaining within the modest range. The remaining biomarkers exhibited consistently lower discriminative capacity across all efficacy domains (Table S1).

#### 3.4.4. Univariate Logistic Regression Analysis of Baseline Biomarkers

For EASI-75 at week 40 (Table S4d), all odds ratios remained non-significant, with the SIRI (OR = 0.55, 95% CI 0.23–1.34, *p* = 0.192; Nagelkerke pseudo-*R*^2^ = 0.06) and LDH (OR = 0.99, 95% CI 0.98–1.00, *p* = 0.194; pseudo-*R*^2^ = 0.07) representing the upper bound of an otherwise undifferentiated predictive field—consistent with their relatively higher AUC values in the ROC analysis (0.63 and 0.66, respectively). For EASI-90 at week 40 (Table S4e), the absolute lymphocyte count yielded the largest pseudo-*R*^2^ (0.07; OR = 2.34, 95% CI 0.68–8.00, *p* = 0.176), yet failed to attain significance. Similarly, for EASI-100 at week 40 (Table S4f), low disease activity at week 40 (Table S4g), and for the week 16 outcomes no predictor reached significance. The complete results are presented in Table S4a–g in Supplementary Materials.

## 4. Discussion

This is the first real-world study to assess the value of inflammatory indices, including SIRI and ELR, alongside ECP, for predicting the short- and long-term clinical response to dupilumab in patients with severe AD. The assessment of the potential predictive performance of *Malassezia*-specific IgE in relation to whole-body treatment response, rather than only facial involvement, is also novel.

We focused on biomarkers that are readily accessible, cost-effective, and potentially applicable in routine clinical practice, while also representing biologically plausible candidates for exploratory investigation. The observed findings represent exploratory associations requiring validation in larger cohorts. Most of the selected biomarkers did not show predictive potential. Nevertheless, these negative findings provide valuable information by helping to refine future research directions and prioritize the most promising biomarker candidates for further evaluation.

When considered collectively across all outcomes and time points, the AUC patterns allow several observations regarding the relative hierarchy and temporal consistency of the examined biomarkers. LDH and SIRI were the most consistently informative biomarkers in the analysis. However, due to the relatively small sample size, the exploratory character of the study, and AUC values not exceeding 0.70, LDH and SIRI should be considered preliminary candidates for future validation rather than established predictive biomarkers. 

Among examined biomarkers, LDH was the most consistently informative continuous baseline biomarker, attaining AUC values ≥ 0.60 for four of the seven outcome combinations (EASI-75 at week 16: 0.60; EASI-90 at week 16: 0.63; EASI-75 at week 40: 0.66; EASI < 7 at week 40: 0.64, respectively). Although none of these values individually surpassed the 0.70 threshold and did not reach statistical significance in logistic regression analyses, the cross-outcome reproducibility of LDH’s discriminative signal—predominantly ranking first or second among continuous baseline predictors—elevates it above all other hematological and biochemical candidates. However, this association should be interpreted with caution, as lower baseline LDH levels were associated with better clinical outcomes in most, but not all, analyses. This finding is consistent with the notion that LDH reflects epidermal turnover and tissue damage, and that patients with higher baseline LDH may harbor a greater inflammatory burden contributing to treatment resistance [18]. Moreover, it aligns with the findings of Kato et al. [16] and Kido-Nakahara et al. [19] who demonstrated a negative association between higher baseline LDH concentrations and clinical improvement over time [16,19].

The systemic inflammatory response index (SIRI) constituted the second most stable potential predictor at baseline, with a lower baseline SIRI favoring a better clinical outcome. Moreover, among the biomarkers assessed at week 16, SIRI demonstrated the highest overall discriminative performance for all evaluated outcomes. SIRI is a novel integrated marker of systemic inflammatory status, showing good predictive value in chronic and acute infection, cancer and stroke [20,21,22]. Its composite nature—integrating neutrophil, monocyte, and lymphocyte signals into a single ratio—may capture a broader dimension of systemic immune status that individual cell counts fail to represent. The composite reduction—driven by downward trends in neutrophils and monocytes coupled with the significant lymphocyte expansion—suggests that dupilumab may be associated with changes in broader innate inflammatory responses beyond the canonical type 2 axis. However, the biological rationale linking SIRI to dupilumab response has not yet been established and requires further studies to clarify whether SIRI reflects pathways relevant to treatment response or represents a nonspecific marker of systemic inflammation. To the best of the authors’ knowledge, SIRI has not yet been evaluated as a predictive biomarker of dupilumab response in adults. It has, however, been studied in relation to upadacitinib [23] and tralokinumab [24], where higher baseline SIRI was observed in primary non-responders to upadacitinib [23], while it did not predict poor response to tralokinumab [24].

Ordinal total serum IgE class did not demonstrate a predictive advantage over the other analyzed parameters. The lack of predictive value is consistent with previous studies, as in the present analysis—and most prior reports [7,25,26,27]—continuous total serum IgE did not demonstrate strong predictive capacity. Notably, to our knowledge, total serum IgE class has not previously been examined in relation to complete skin clearance and long-term disease control, and therefore warrants further investigation in larger cohorts.

Moreover, Malassezia-specific IgE did not demonstrate coherent or replicable discriminative performance. Although it has previously been proposed as a potential outcome biomarker in patients with atopic dermatitis undergoing dupilumab therapy, the existing evidence has primarily focused on its association with dupilumab-associated head and neck dermatitis [14]. In the present study, we evaluated its potential as a biomarker of overall treatment response and did not find evidence supporting its utility.

The remaining biomarkers—WBC count, lymphocyte count, monocyte count, neutrophil count, absolute eosinophil count, eosinophil percentage, ELR—exhibited sporadic, non-replicable elevations in AUC that did not persist across outcomes or time points, and therefore cannot be regarded as reliable standalone predictors in this cohort. Similar findings were reported elsewhere [7]. Serum ECP, which increased numerically during treatment and showed directional concordance with the eosinophil trends consistent with enhanced peripheral eosinophil activation during therapy (though the wide confidence interval reflected substantial inter-individual variability), also did not demonstrate clinically useful discriminative capacity.

The study has several limitations. First, the sample size was relatively small, with imbalance between responder and non-responder groups for more stringent endpoints, which may limit the statistical power and generalizability of the findings; however, considering the single-center prospective design, the cohort size remains relatively substantial. Nevertheless, analyses should be interpreted as exploratory, particularly in more stringent outcome categories. Second, disease activity may have been influenced by factors not fully controlled for, including suboptimal adherence to emollient therapy and environmental exposures. Additionally, concomitant medications prescribed for comorbid conditions could have affected inflammatory parameters or clinical outcomes; however, in accordance with the exclusion criteria, none of the patients were receiving systemic glucocorticosteroids at the time of blood sampling. Moreover, patients characteristics, such as age, sex, comorbidities (especially potential hematological disorders [28], which, to our knowledge, were not reported in the study population) or prior treatment, may have influenced treatment response. Patient oriented scores were not analyzed, as the study aimed to focus on objective treatment results; however, further research could also consider patient-reported outcome measures. Finally, the study population consisted exclusively of Polish patients recruited at a single center, which may limit the external validity of the results to other ethnic or geographic groups. Reliable multivariable modeling was not feasible due to the limited sample size.

## 5. Conclusions

In aggregate, LDH and SIRI emerge as the most promising candidates for further investigation in larger cohorts, owing to their cross-outcome and cross-time point consistency rather than to any single dominant AUC value. No single baseline laboratory biomarker provides reliable, clinically actionable discriminative capacity for any dupilumab response endpoint, including complete skin clearance (EASI-100), in this cohort of patients with severe atopic dermatitis. The discriminative potential of individual biomarkers, although detectable, remained below the threshold of clinical utility throughout. The possibility that composite multi-biomarker panels—rather than individual markers—may be required to achieve clinically meaningful predictive accuracy warrants dedicated exploration in future studies.

## Figures and Tables

**Table 1:** Baseline demographic, clinical, and laboratory characteristics of the study cohort (*n* = 40).

Characteristic	Value	95% CI
I. Demographics		
Age (years)	32.5 (25.0–41.0)	27.0–40.0
Sex		
Male	26 (65.0%)	49.5–77.9
Female	14 (35.0%)	22.1–50.5
Age at onset (years)	1.0 (1.0–7.0)	1.0–5.0
Disease duration (years)	27.0 (19.6–38.3)	23.0–31.0
Baseline EASI score	26.5 (22.0–38.4)	22.4–33.2
II. Comorbidities		
Allergic rhinitis	31 (77.5%)	62.5–87.7
Asthma	18 (45.0%)	30.7–60.2
Hypothyroidism	6 (15.0%)	7.1–29.1
Other autoimmune diseases	3 (7.5%)	2.6–19.9
Depression	5 (12.5%)	5.5–26.4
III. Prior systemic therapy		
Any prior systemic therapy	33 (82.5%)	68.1–91.3
Cyclosporine A	33 (82.5%)	68.1–91.3
Methotrexate	4 (10.0%)	4.0–23.1
Phototherapy	5 (12.5%)	5.5–26.4
Tralokinumab	2 (5.0%)	1.4–16.5
Nemolizumab	1 (2.5%)	0.4–12.9
Treatment-naïve	7 (17.5%)	8.7–31.9

Notes: Continuous variables are presented as median (Q1–Q3). Categorical variables are presented as *n* (%). 95% confidence intervals for categorical proportions were calculated using the Wilson score method. 95% confidence intervals for continuous variables are distribution-free intervals for the median (order-statistic method). Abbreviations: EASI, Eczema Area and Severity Index.

**Table 2:** Longitudinal comparison of laboratory parameters from baseline to week 16 of dupilumab therapy.

Variable	Baseline (*n* = 40)	Baseline 95% CI	Week 16 (*n* = 40)	Week 16 95% CI	Median Change (95% CI)	*p*-Value *
**I. Hematological parameters**						
White blood cell count (×10^3^/μL)	6.53 (5.57–7.26)	6.16–7.03	6.55 (5.51–7.80)	5.90–6.93	−0.10 (−0.63 to 0.19)	0.727
Absolute lymphocyte count (×10^3^/μL)	1.58 (1.34–2.03)	1.38–1.76	1.75 (1.42–2.18)	1.57–2.09	0.17 (0.05 to 0.30)	**0.006**
Absolute neutrophil count (×10^3^/μL)	3.71 (2.92–4.52)	3.16–4.24	3.58 (2.57–4.21)	2.66–3.88	−0.32 (−0.73 to 0.10)	0.195
Absolute monocyte count (×10^3^/μL)	0.59 (0.51–0.70)	0.54–0.66	0.60 (0.48–0.67)	0.53–0.63	−0.03 (−0.09 to 0.04)	0.503
Absolute eosinophil count (×10^3^/μL)	0.43 (0.28–0.61)	0.33–0.55	0.42 (0.27–0.95)	0.31–0.74	0.12 (−0.01 to 0.29)	0.072
Eosinophil (%)	6.90 (4.40–8.80)	5.60–7.80	7.10 (4.50–13.75)	5.30–10.60	1.50 (−0.25 to 3.85)	0.096
**II. Composite inflammatory indices**						
Eosinophil-to-lymphocyte ratio (ELR)	0.26 (0.17–0.44)	0.21–0.34	0.30 (0.15–0.49)	0.17–0.40	0.02 (−0.05 to 0.11)	0.476
Systemic inflammatory response index (SIRI)	1.31 (1.05–1.81)	1.11–1.57	1.16 (0.76–1.70)	0.79–1.53	−0.30 (−0.56 to −0.07)	**0.012**
**III. Biochemical markers**						
Lactate dehydrogenase (LDH, U/L) ^a^	236.0 (208.0–286.0)	216–277	182.0 (171.0–202.0)	172–193	−54.5 (−75.0 to −37.5)	**<0.001**
Eosinophil cationic protein (ECP, μg/L)	18.55 (12.85–31.80)	14.6–27.5	23.20 (15.00–40.85)	17.4–28.6	3.96 (−0.75 to 16.50)	0.097
**IV. Immunoglobulin E parameters**						
Total serum IgE (kU/L)	3439.5 (201.5–5000.0)	748–5000	1698.5 (96.3–3806.0)	148–2577	−874.0 (−1388.0 to −480.0)	**<0.001**
Total serum IgE class					–	0.731
Class 0	8 (20.0%)	9.6–36.0	11 (27.5%)	15.0–44.0	–	–
Class I–II	8 (20.0%)	9.6–36.0	7 (17.5%)	7.9–33.0	–	–
Class III–IV	24 (60.0%)	43.0–75.0	22 (55.0%)	39.0–70.0	–	–
Malassezia-specific IgE (kUA/L)	4.10 (0.26–16.15)	3.8–16.0	2.41 (0.16–7.47)	2.1–9.6	−3.26 (−6.22 to −1.22)	**<0.001**
Malassezia-specific IgE class					–	0.617
Class 0–II	19 (47.5%)	32.0–64.0	23 (57.5%)	41.0–73.0	–	–
Class III–IV	19 (47.5%)	32.0–64.0	16 (40.0%)	25.0–57.0	–	–
Class V–VI	2 (5.0%)	0.9–18.0	1 (2.5%)	0.1–15.0	–	–

Notes: ^a^ *n* = 39 (one missing value). * *p*-values for continuous and ordinal variables were calculated using the Wilcoxon signed-rank test for paired samples (two-sided). For categorical IgE class distributions the Stuart–Maxwell test of marginal homogeneity was used. Continuous variables are presented as median (Q1–Q3). Categorical variables are presented as *n* (%). 95% confidence intervals for continuous variables are distribution-free intervals for the median (order-statistic method) for proportions, Wilson score method; for median change, Hodges–Lehmann estimator. Abbreviations: ECP, eosinophilic cationic protein; ELR, eosinophil-to-lymphocyte ratio; IQR, interquartile range; LDH, lactate dehydrogenase; SIRI, systemic inflammatory response index.

**Table 3:** Clinical response to dupilumab therapy as assessed by the Eczema Area and Severity Index score and percentage reduction from baseline (*n* = 40).

Variable	Baseline (*n* = 40)	Baseline 95% CI	Week 16 (*n* = 40)	Week 16 95% CI	Week 40 (*n* = 39)	Week 40 95% CI	*p*-Value
EASI score	26.50 (22.00–38.55) [20.20, 65.10]	22.4–33.2	4.10 (2.15–7.77) [0, 18.70]	2.6–5.8	2.00 (0.50–7.80) [0, 21.90]	0.6–5.3	**<0.001 ^1^**
Percentage reduction in EASI score from baseline (%)	—	—	84.16 (73.92–93.11) [51.60, 100.00]	78.0–88.0	92.80 (73.44–99.08) [12.75, 100.00]	81.0–95.0	0.265 ^2^

Notes: ^1^ *p*-value obtained from the Friedman test (non-parametric repeated-measures analysis across the three time points). ^2^ *p*-value obtained from Wilcoxon signed-rank test for paired samples (two-sided, comparison between week 16 and week 40, change 3.27%, CI: −2.8% to 9.1%). All values are presented as median (Q1–Q3) [min, max]. 95% confidence intervals are displayed in dedicated columns. 95% confidence intervals for the EASI score are distribution-free intervals for the median (order-statistic method); percentage reductions are derived from paired individual patient data. Abbreviations: EASI, Eczema Area and Severity Index; IQR, interquartile range.

**Table 4:** Achievement of EASI response thresholds and low disease activity at weeks 16 and 40 of dupilumab therapy (*n* = 40).

Variable	Week 16 *n* (%), (*n* = 40)	Week 16 95% CI	Week 40 *n* (%), (*n* = 39)	Week 40 95% CI	*p*-Value *
Achievement of EASI-75 response	29 (72.5%)	56.6–84.6	29 (74.3%)	58.0–86.0	1.000
Achievement of EASI-90 response	14 (35.0%)	21.7–51.0	24 (61.5%)	45.0–76.0	**0.018**
Achievement of EASI-100 response	1 (2.5%)	0.1–13.9	9 (23.1%)	12.0–40.0	**0.030**
EASI score < 7 (low disease activity)	28 (70.0%)	53.6–82.6	28 (71.8%)	55.0–84.0	1.000

Notes *—*p*-values were calculated using McNemar’s test for paired proportions (two-sided). Categorical variables are presented as *n* (%). 95% confidence intervals for proportions were calculated using the Wilson score method. Abbreviations: EASI, Eczema Area and Severity Index.

**Table 5:** Predictive performance of baseline laboratory biomarkers for clinical response to dupilumab at week 16: receiver-operating characteristic (ROC) analysis (optimal cut-off by Youden index). *For 95% confidence intervals (CIs) for AUC, PPV and NPV*, see Supplementary Table S2.

Variable	Cutpoint	Youden	Accuracy	Sensitivity	Specificity	AUC
**I. Achievement of EASI-75 response**						
Hematological parameters						
White blood cell count (×10^3^/μL)	≥5.14	0.20	0.75	0.93	0.27	0.53
Absolute lymphocyte count (×10^3^/μL)	≥1.42	0.33	0.68	0.69	0.63	0.57
Absolute neutrophil count (×10^3^/μL)	≥2.88	0.32	0.75	0.86	0.45	0.59
Absolute monocyte count (×10^3^/μL)	≤0.64	0.14	0.63	0.69	0.45	0.50
Absolute eosinophil count (×10^3^/μL)	≥0.48	0.21	0.55	0.48	0.73	0.55
Eosinophil (%)	≥6.70	0.13	0.58	0.59	0.55	0.52
Composite inflammatory indices						
Eosinophil-to-lymphocyte ratio (ELR)	≥0.35	0.20	0.50	0.38	0.82	0.55
Systemic inflammatory response index (SIRI)	≥1.11	0.18	0.65	0.72	0.45	0.52
Biochemical markers						
Lactate dehydrogenase (LDH, U/L) ^a^	≥284.00	0.27	0.51	0.36	0.91	0.60
Eosinophil cationic protein (ECP, μg/L)	≤9.28	0.14	0.38	0.14	1.00	0.50
Immunoglobulin E parameters						
Total serum IgE (kU/L)	≤2810.00	0.15	0.55	0.52	0.64	0.56
Malassezia-specific IgE (kUA/L)	≤0.06	0.21	0.43	0.21	1.00	0.52
**II. Achievement of EASI-90 response**						
Hematological parameters						
White blood cell count (×10^3^/μL)	≤6.89	0.29	0.60	0.79	0.50	0.53
Absolute lymphocyte count (×10^3^/μL)	≥1.87	0.20	0.65	0.43	0.77	0.54
Absolute neutrophil count (×10^3^/μL)	≥2.82	0.27	0.53	1.00	0.27	0.52
Absolute monocyte count (×10^3^/μL)	≥0.59	0.22	0.60	0.64	0.58	0.55
Absolute eosinophil count (×10^3^/μL)	≥0.69	0.06	0.63	0.21	0.85	0.56
Eosinophil (%)	≥6.10	0.18	0.55	0.71	0.46	0.56
Composite inflammatory indices						
Eosinophil-to-lymphocyte ratio (ELR)	≥0.51	0.24	0.70	0.36	0.88	0.58
Systemic inflammatory response index (SIRI)	≤1.05	0.16	0.65	0.36	0.81	0.51
Biochemical markers						
Lactate dehydrogenase (LDH, U/L) ^a^	≤204.00	0.31	0.72	0.43	0.88	0.63
Eosinophil cationic protein (ECP, μg/L)	≥28.90	0.27	0.68	0.50	0.77	0.60
Immunoglobulin E parameters						
Total serum IgE (kU/L)	≤258.00	0.24	0.68	0.43	0.81	0.54
Malassezia-specific IgE (kUA/L)	≤4.99	0.21	0.58	0.71	0.50	0.58
**III. Achievement of EASI score < 7 (low disease activity)**						
Hematological parameters						
White blood cell count (×10^3^/μL)	≤6.89	0.38	0.70	0.71	0.67	0.67
Absolute lymphocyte count (×10^3^/μL)	≤2.18	0.26	0.75	0.93	0.33	0.58
Absolute neutrophil count (×10^3^/μL)	≤4.35	0.32	0.73	0.82	0.50	0.56
Absolute monocyte count (×10^3^/μL)	≤0.64	0.33	0.70	0.75	0.58	0.66
Absolute eosinophil count (×10^3^/μL)	≥0.59	−0.05	0.40	0.29	0.67	0.52
Eosinophil (%)	≥6.2	0.14	0.60	0.64	0.50	0.55
Composite inflammatory indices						
Eosinophil-to-lymphocyte ratio (ELR)	≥0.35	0.35	0.58	0.43	0.92	0.61
Systemic inflammatory response index (SIRI)	≤1.75	0.24	0.70	0.82	0.42	0.54
Biochemical markers						
Lactate dehydrogenase (LDH, U/L) ^a^	≤218.00	0.28	0.56	0.44	0.82	0.58
Eosinophil cationic protein (ECP, μg/L)	≤19.50	0.39	0.68	0.64	0.75	0.61
Immunoglobulin E parameters						
Total serum IgE (kU/L)	≤258.00	0.39	0.58	0.39	1.00	0.67
Malassezia-specific IgE (kUA/L)	≤4.12	0.12	0.55	0.54	0.58	0.63

Notes: ^a^ Available for 39 patients (one missing value). Optimal cut-off determined by maximizing the Youden index (sensitivity + specificity − 1). All analyses performed on complete cases only. Abbreviations: AUC, area under the receiver-operating characteristic curve; EASI, Eczema Area and Severity Index; ECP, eosinophilic cationic protein; ELR, eosinophil-to-lymphocyte ratio; LDH, lactate dehydrogenase; NPV, negative predictive value; SIRI, systemic inflammatory response index, PPV, positive predictive value.

**Table 6:** Diagnostic performance of baseline IgE class variables for predicting EASI response at week 16. *For 95% confidence intervals (CIs) for AUC, PPV and NPV*, see Supplementary Table S2.

Variable	Outcome	*n*	Accuracy	Sensitivity	Specificity	PPV	NPV	AUC
**I. Achievement of EASI-75** **response**								
Total serum IgE class	EASI-75 at Week 16	40	0.53	0.59	0.36	0.71	0.25	0.47
Malassezia-specific IgE class	EASI-75 at Week 16	40	0.50	0.52	0.45	0.71	0.26	0.50
**II. Achievement of EASI-90** **response**								
Total serum IgE class	EASI-90 at Week 16	40	0.45	0.57	0.38	0.33	0.62	0.48
Malassezia-specific IgE class	EASI-90 at Week 16	40	0.42	0.43	0.42	0.29	0.58	0.44
**III. Achievement of EASI score < 7**								
Total serum IgE class	EASI < 7 at Week 16	40	0.45	0.54	0.25	0.62	0.19	0.36
Malassezia-specific IgE class	EASI < 7 at Week 16	40	0.42	0.46	0.33	0.62	0.21	0.42

Notes: Positive test result was defined as membership in the elevated IgE class tier (Class III–IV for total serum IgE; Class III–VI, i.e., Classes III–IV and V–VI combined, for Malassezia-specific IgE). Outcome-positive class = “Yes” for all binary outcomes. AUC was computed with the IgE class treated as an ordinal numeric variable, oriented so that a higher class denotes a higher predicted probability of response; 95% confidence intervals for the ordinal IgE-class AUC were obtained by stratified bootstrap (2000 resamples). All analyses were complete-case (*n* = 40). Every AUC 95% confidence interval included 0.50, indicating no discriminative ability for either IgE-class variable. Abbreviations: AUC, area under the receiver-operating characteristic curve; EASI, Eczema Area and Severity Index; NPV, negative predictive value; PPV, positive predictive value.

**Table 7:** Predictive performance of baseline laboratory biomarkers for clinical response to dupilumab at week 40: receiver-operating characteristic (ROC) analysis (optimal cut-off by Youden index). *For 95% confidence intervals (CIs) for AUC, PPV and NPV*, see Supplementary Table S2.

Variable	Cutpoint	Youden	Accuracy	Sensitivity	Specificity	AUC
**I. Achievement of EASI-75 response**						
Hematological parameters						
White blood cell count (×10^3^/μL)	≤5.89	0.18	0.49	0.40	0.80	0.58
Absolute lymphocyte count (×10^3^/μL)	≥1.87	0.28	0.51	0.38	0.90	0.59
Absolute neutrophil count (×10^3^/μL)	≤4.65	0.26	0.74	0.86	0.40	0.60
Absolute monocyte count (×10^3^/μL)	≥0.72	0.18	0.44	0.28	0.90	0.51
Absolute eosinophil count (×10^3^/μL)	≤0.47	0.25	0.64	0.66	0.60	0.51
Eosinophil (%)	≥2.70	0.17	0.77	0.97	0.20	0.50
Composite inflammatory indices						
Eosinophil-to-lymphocyte ratio (ELR)	≥0.08	0.20	0.79	1.00	0.20	0.51
Systemic inflammatory response index (SIRI)	≤1.86	0.36	0.77	0.86	0.50	0.63
Biochemical markers						
Lactate dehydrogenase (LDH, U/L) ^a^	≤238.00	0.38	0.68	0.68	0.70	0.66
Eosinophil cationic protein (ECP, μg/L)	≥11.1	0.23	0.77	0.93	0.30	0.52
Immunoglobulin E parameters						
Total serum IgE (kU/L)	≤260.00	0.28	0.51	0.38	0.90	0.58
Malassezia-specific IgE (kUA/L)	≤0.32	0.38	0.54	0.38	1.00	0.64
**II. Achievement of EASI-90 response**						
Hematological parameters						
White blood cell count (×10^3^/μL)	≥4.79	0.16	0.67	0.96	0.20	0.53
Absolute lymphocyte count (×10^3^/μL)	≥1.87	0.28	0.59	0.42	0.87	0.62
Absolute neutrophil count (×10^3^/μL)	≥3.05	0.19	0.64	0.79	0.40	0.55
Absolute monocyte count (×10^3^/μL)	≤0.59	0.23	0.62	0.63	0.60	0.57
Absolute eosinophil count (×10^3^/μL)	≤0.47	0.31	0.67	0.71	0.60	0.57
Eosinophil (%)	≤6.80	0.29	0.64	0.63	0.67	0.60
Composite inflammatory indices						
Eosinophil-to-lymphocyte ratio (ELR)	≤0.17	0.24	0.56	0.38	0.87	0.58
Systemic inflammatory response index (SIRI)	≤1.28	0.29	0.64	0.63	0.67	0.59
Biochemical markers						
Lactate dehydrogenase (LDH, U/L) ^a^	≤238.00	0.19	0.61	0.65	0.53	0.57
Eosinophil cationic protein (ECP, μg/L)	≤14.4	0.22	0.56	0.42	0.80	0.52
Immunoglobulin E parameters						
Total serum IgE (kU/L)	≤260.0	0.18	0.54	0.38	0.80	0.53
Malassezia-specific IgE (kUA/L)	≥12.3	0.18	0.54	0.38	0.80	0.50
**III. Achievement of EASI-100 response**						
Hematological parameters						
White blood cell count (×10^3^/μL)	≥5.36	0.14	0.64	0.44	0.70	0.51
Absolute lymphocyte count (×10^3^/μL)	≥1.87	0.18	0.67	0.44	0.73	0.54
Absolute neutrophil count (×10^3^/μL)	≥2.88	0.30	0.46	1.00	0.30	0.55
Absolute monocyte count (×10^3^/μL)	≤0.46	0.31	0.77	0.44	0.87	0.59
Absolute eosinophil count (×10^3^/μL)	≤0.18	0.23	0.77	0.33	0.90	0.59
Eosinophil (%)	≤9.1	0.27	0.44	1.00	0.27	0.59
Composite inflammatory indices						
Eosinophil-to-lymphocyte ratio (ELR)	≤0.31	0.07	0.46	0.67	0.40	0.61
Systemic inflammatory response index (SIRI)	≤1.21	0.33	0.67	0.67	0.67	0.60
Biochemical markers						
Lactate dehydrogenase (LDH, U/L) ^a^	≤238.00	0.26	0.56	0.78	0.48	0.59
Eosinophil cationic protein (ECP, μg/L)	≤29.30	0.19	0.44	0.89	0.30	0.54
Immunoglobulin E parameters						
Total serum IgE (kU/L)	≤260.00	0.18	0.67	0.44	0.73	0.54
Malassezia-specific IgE (kUA/L)	≥3.91	0.20	0.56	0.67	0.53	0.53
**IV. Achievement of EASI score < 7 (low disease activity)**						
Hematological parameters						
White blood cell count (×10^3^/μL)	≤7.74	0.13	0.69	0.86	0.27	0.52
Absolute lymphocyte count (×10^3^/μL)	≥1.87	0.30	0.54	0.39	0.91	0.62
Absolute neutrophil count (×10^3^/μL)	≤4.65	0.22	0.72	0.86	0.37	0.53
Absolute monocyte count (×10^3^/μL)	≤0.44	0.18	0.41	0.18	1.00	0.55
Absolute eosinophil count (×10^3^/μL)	≥0.17	0.15	0.74	0.96	0.18	0.50
Eosinophil (%)	≤6.80	0.21	0.59	0.57	0.64	0.51
Composite inflammatory indices						
Eosinophil-to-lymphocyte ratio (ELR)	≤0.27	0.15	0.59	0.61	0.55	0.50
Systemic inflammatory response index (SIRI)	≤1.28	0.33	0.64	0.61	0.73	0.64
Biochemical markers						
Lactate dehydrogenase (LDH, U/L) ^a^	≤218.00	0.39	0.61	0.48	0.91	0.64
Eosinophil cationic protein (ECP, μg/L)	≥11.1	0.20	0.74	0.93	0.27	0.51
Immunoglobulin E parameters						
Total serum IgE (kU/L)	≤260.00	0.30	0.54	0.39	0.91	0.58
Malassezia-specific IgE (kUA/L)	≤0.32	0.27	0.51	0.36	0.91	0.59

Notes: ^a^ Available for 39 patients (one missing value). Optimal cut-off determined by maximising the Youden index (sensitivity + specificity − 1). All analyses performed on complete cases only. Abbreviations: AUC, area under the receiver-operating characteristic curve; EASI, Eczema Area and Severity Index; ECP, eosinophilic cationic protein; ELR, eosinophil-to-lymphocyte ratio; LDH, lactate dehydrogenase; NPV, negative predictive value; PPV, positive predictive value, SIRI, systemic inflammatory response index.

**Table 8:** Diagnostic performance of baseline IgE class variables for predicting EASI response at week 40 (highest class considered as positive test result). *For 95% confidence intervals (CIs) for AUC*, *PPV and NPV* see Supplementary Table S3.

Variable	Outcome	*n*	Accuracy	Sensitivity	Specificity	PPV	NPV	AUC
**I. Achievement of EASI-75 response**								
Total serum IgE class	EASI-75 at Week 40	39	0.49	0.55	0.30	0.70	0.19	0.41
Malassezia-specific IgE class	EASI-75 at Week 40	39	0.46	0.48	0.40	0.70	0.21	0.42
**II. Achievement of EASI-90 response**								
Total serum IgE class	EASI-90 at Week 40	39	0.46	0.54	0.33	0.57	0.31	0.45
Malassezia-specific IgE class	EASI-90 at Week 40	39	0.54	0.54	0.53	0.65	0.42	0.52
**III. Achievement of EASI-100 response**								
Total serum IgE class	EASI-100 at Week 40	39	0.44	0.56	0.40	0.22	0.75	0.48
Malassezia-specific IgE class	EASI-100 at Week 40	39	0.56	0.67	0.53	0.30	0.84	0.59
**IV. Achievement of EASI < 7**								
Total serum IgE class	EASI < 7 at Week 40	39	0.46	0.54	0.27	0.65	0.19	0.39
Malassezia-specific IgE class	EASI < 7 at Week 40	39	0.49	0.50	0.45	0.70	0.26	0.45

Notes: Positive test result was defined as membership in the elevated IgE class tier (Class III–IV for total serum IgE; Class III–VI, i.e., Classes III–IV and V–VI combined, for Malassezia-specific IgE). Outcome-positive class = “Yes” for all binary outcomes. AUC was computed with the IgE class treated as an ordinal numeric variable, oriented so that a higher class denotes a higher predicted probability of response; 95% confidence intervals for the ordinal IgE-class AUC were obtained by stratified bootstrap (2000 resamples). Analyses were complete-case (*n* = 39; one participant lacked a Week-40 outcome). Every AUC 95% confidence interval included 0.50, indicating no discriminative ability for either IgE-class variable. Abbreviations: AUC, area under the receiver-operating characteristic curve; EASI, Eczema Area and Severity Index; NPV, negative predictive value; PPV, positive predictive value.

## Data Availability

The original contributions presented in this study are included in the article and Supplementary Materials. Further inquiries can be directed to the corresponding author.

## Supplementary Materials

The following supporting information can be downloaded at: https://www.mdpi.com/article/10.3390/biomedicines14081767/s1. Supplementary Material S1: Statistical analysis details; Table S1a: Predictive performance of week 16 laboratory biomarkers for clinical response to dupilumab at week 40: receiver-operating characteristic (ROC) analysis (optimal cut-off by Youden index); Table S1b: Diagnostic performance of week 16 IgE class variables for predicting EASI response at week 40 (highest class considered as positive test result); Table S2a: ROC discrimination of baseline continuous biomarkers at week 16, with 95% CIs; Table S2b: ROC discrimination of baseline IgE class variables at week 16, with 95% CIs); Table S3a: ROC discrimination of baseline continuous biomarkers at week 40, with 95% Cis; Table S3b: ROC discrimination of baseline IgE class variables at week 40, with 95% Cis; Table S4a: Univariate logistic regression: baseline predictors of EASI-75 (Week 16); Table S4b: Univariate logistic regression: baseline predictors of EASI-90 (Week 16); Table S4c: Univariate logistic regression: baseline predictors of EASI < 7 (Week 16); Table S4d: Univariate logistic regression: baseline predictors of EASI-75 (Week 40); Table S4e: Univariate logistic regression: baseline predictors of EASI-90 (Week 40); Table S4f: Univariate logistic regression: baseline predictors of EASI-100 (Week 40); Table S4g: Univariate logistic regression: baseline predictors of EASI < 7 (Week 40); Figure S1: Participant flow.

## Abbreviations

The following abbreviations are used in this manuscript:

AD	Atopic dermatitis
AUC	Area under the receiver-operating characteristic curve
CI	Confidence interval
EASI	Eczema Area and Severity Index
EASI75	at least 75% improvement in the Eczema Area and Severity Index
EASI90	at least 90% improvement in the Eczema Area and Severity Index
EASI100	100% improvement in the Eczema Area and Severity Index
ECP	Eosinophilic cationic protein
ELR	Eosinophil-to-lymphocyte ratio
IQR	Interquartile range
IL-4	Interleukin 4
IL-13	Interleukin 13
LDH	Lactate dehydrogenase
NPV	Negative predictive value
PPV	Positive predictive value
SD	Standard deviation
SIRI	Systemic inflammatory response index

## Appendix A. Software and Reproducibility

Analyses were conducted using the R packages (version 4.5.2) *ggalluvial* [29], *flextable* [30], *rstatix* [31], *tibble* [32], *patchwork* [33], *PMCMRplus* [34], *broom* [35], *gtsummary* [36], *stringr* [37], *dplyr* [38], *scales* [39], *tidyr* [40], *pROC* [41], *coin* [42], *ggplot2* [43], *forcats* [44], *cutpointr* [45], *car* [46], *report* [47].
