# Supplementary material for: Evaluation of Selected Laboratory Parameters as Predictive Biomarkers of Clinical Response to Dupilumab Therapy in Patients with Atopic Dermatitis
Source: Biomedicines. 2026 Aug 6;14(8):1767. doi: 10.3390/biomedicines14081767 (PMC13510145; doi:10.3390/biomedicines14081767)
Supplement: Supplementary file 1 [file biomedicines-14-01767-s001.zip › XX Supplementary_Material_revised_tracked 08.26 (1) proof.pdf]

Supplementary Material S1

Statistical Analysis Details

Predictor Variables

Total serum IgE exceeded the upper assay quantification limit of 5,000 kU/L in 16 of 40 patients (40.0%), while one value fell below the lower detection threshold (<2 kU/L). To avoid substitution-dependent inference, the ordinal IgE class was the pre-specified representation for the logistic-regression analyses. The rank-based analyses (Wilcoxon signed-rank test and the area under the ROC curve) are invariant to the ceiling substitution, because every censored value exceeds the maximum uncensored value (4,173 kU/L); the continuous form was therefore used only for those rank-based displays and as a labelled sensitivity analysis. The ordinal class (class 0 through 4) is defined as follows: Class 0 (0-100 kU/L); Class 1 (101-300 kU/L); Class 2 (301-1000 kU/L); Class 3 (1001-5000 kU/L); Class 4 (>5000 kU/L).

Malassezia-specific IgE, which did not exhibit systematic censoring, was analysed both in its native continuous form (kUA/L) and as an ordinal class (0–6) to permit a sensitivity comparison between continuous and categorical operationalisations. The classes were ordered as follows: Class 0 (0-0,34 kUA/L); Class 1 (0,35-0,69 kUA/L); Class 2 (0,70-3,49 kUA/L); Class 3 (3,50-17,49 kUA/L); Class 4 (17,50-49,99 kUA/L); Class 5 (50,00-100,0 kUA/L); Class 6 (>100,0 kUA/L).

Clinical Outcome Variables

Clinical outcomes were defined as dichotomous variables (responder versus non-responder) at two assessment time points. At week 16, three outcomes were evaluated: EASI-75, EASI-90, and low disease activity (EASI < 7). The EASI-100 outcome at week 16 comprised only a single responder (1/40; 2.5%), rendering formal ROC analysis unreliable; this endpoint was therefore excluded from predictive modelling at week 16 and retained only for descriptive and alluvial analyses. At week 40, four outcomes were evaluated: EASI-75, EASI-90, EASI-100 (9 responders; 23.1%, deemed sufficient for meaningful evaluation), and EASI < 7.

Longitudinal Trajectory of EASI Scores

The temporal trajectory of EASI scores across the three measurement time points (baseline, week 16, week 40) was assessed using the Friedman non-parametric repeated-measures test, with Kendall's coefficient of concordance (W) and its 95% confidence interval reported as a measure of effect consistency. Where the omnibus Friedman test was significant, pairwise post hoc comparisons were conducted using the Durbin–Conover procedure with Holm adjustment for multiple testing. The percentage reduction in EASI from baseline at weeks 16 and 40 was computed on a per-patient basis and compared between the two on-treatment time points using the Wilcoxon signed-rank test.

Temporal Stability of Categorical Response

The temporal stability of categorical response status between weeks 16 and 40 was examined using McNemar's test for paired proportions, with corresponding odds ratios and 95% confidence intervals. A two-sided P value < 0.05 was considered indicative of statistical significance throughout all analyses.

Receiver Operating Characteristic (ROC) Curve Analysis

ROC curve analysis constituted the principal method for evaluating the discriminative capacity of individual baseline laboratory parameters with respect to each binary clinical outcome. Discrimination is reported as the area under the ROC curve (AUC) with its 95% confidence interval (DeLong method for continuous predictors; stratified bootstrap with 2,000 resamples for the ordinal IgE-class predictors).The area under the ROC curve (AUC) was computed as a global measure of discriminatory ability (0.5 = chance; 1.0 = perfect discrimination). For week 16 outcomes (EASI-75, EASI-90, EASI < 7), baseline values of all continuous biomarkers and ordinal IgE class variables were evaluated as candidate predictors. For week 40 outcomes (EASI-75, EASI-90, EASI-100, EASI < 7), baseline values were likewise evaluated. Predictive performance of week 16 laboratory biomarkers for clinical response to dupilumab at week 40 was also assessed (Table S1a, Table S1b). Ordinal IgE class variables (total serum IgE class 0–4; *Malassezia*-specific IgE class 0–6) were not treated as continuous predictors. Instead, each successive class boundary was evaluated as a potential discriminative threshold within the ROC framework. For the purpose of defining the positive test result, the highest severity categories were designated as the positive class, consistent with the clinical rationale that more intense allergic sensitisation may modify treatment response.
The optimal discriminative threshold for each candidate biomarker was determined using the Youden index (J = sensitivity + specificity − 1). For each optimal cut-off, sensitivity, specificity, positive predictive value (PPV), negative predictive value (NPV), and corresponding 95% confidence intervals were reported. These intervals are provided throughout the ROC tables: the AUC interval by the DeLong method for continuous predictors and by stratified bootstrap for the ordinal classes, and the sensitivity, specificity, PPV and NPV intervals by the exact binomial (Clopper–Pearson) method. Youden-derived cut-offs and their sensitivity and specificity were optimised in-sample and are therefore presented as exploratory. A fixed random seed was used for all resampling.

Table S1a. Predictive performance of week 16 laboratory biomarkers for clinical response to dupilumab at week 40: receiver-operating characteristic (ROC) analysis (optimal cut-off by Youden index).

I. Achievement of EASI-75 response (week 40)

Predictor	Cut-off	AUC (95% CI)	Sensitivity (95% CI)	Specificity (95% CI)	PPV (95% CI)	NPV (95% CI)
White blood cell count	9.23	0.50 (0.26–0.74)	0.97 (0.82–1.00)	0.20 (0.03–0.56)	0.78 (0.61–0.90)	0.67 (0.09–0.99)
Absolute lymphocyte count	2.13	0.56 (0.36–0.76)	0.38 (0.21–0.58)	0.90 (0.55–1.00)	0.92 (0.62–1.00)	0.33 (0.17–0.54)
Absolute neutrophil count	6.15	0.55 (0.31–0.79)	1.00 (0.88–1.00)	0.20 (0.03–0.56)	0.78 (0.62–0.90)	1.00 (0.16–1.00)
Absolute monocyte count	0.54	0.55 (0.35–0.74)	0.41 (0.24–0.61)	0.80 (0.44–0.97)	0.86 (0.57–0.98)	0.32 (0.15–0.54)
Absolute eosinophil count	0.60	0.56 (0.36–0.76)	0.45 (0.26–0.64)	0.80 (0.44–0.97)	0.87 (0.60–0.98)	0.33 (0.16–0.55)
Eosinophil	6.65	0.44 (0.23–0.65)	0.52 (0.33–0.71)	0.60 (0.26–0.88)	0.79 (0.54–0.94)	0.30 (0.12–0.54)
Eosinophil-to-lymphocyte ratio	0.36	0.54 (0.33–0.76)	0.48 (0.29–0.67)	0.70 (0.35–0.93)	0.82 (0.57–0.96)	0.32 (0.14–0.55)
Systemic inflammatory response index	1.18	0.65 (0.45–0.85)	0.66 (0.46–0.82)	0.70 (0.35–0.93)	0.86 (0.65–0.97)	0.41 (0.18–0.67)
Lactate dehydrogenase	196.00	0.56 (0.37–0.76)	0.36 (0.19–0.56)	0.90 (0.55–1.00)	0.91 (0.59–1.00)	0.33 (0.17–0.54)
Eosinophil cationic protein	25.60	0.54 (0.32–0.75)	0.45 (0.26–0.64)	0.70 (0.35–0.93)	0.81 (0.54–0.96)	0.30 (0.13–0.53)
Total serum IgE	125.50	0.57 (0.35–0.78)	0.38 (0.21–0.58)	0.90 (0.55–1.00)	0.92 (0.62–1.00)	0.33 (0.17–0.54)
Malassezia-specific IgE	2.62	0.63 (0.44–0.83)	0.62 (0.42–0.79)	0.70 (0.35–0.93)	0.86 (0.64–0.97)	0.39 (0.17–0.64)

II. Achievement of EASI-90 response (week 40)

Predictor	Cut-off	AUC (95% CI)	Sensitivity (95% CI)	Specificity (95% CI)	PPV (95% CI)	NPV (95% CI)
White blood cell count	5.24	0.51 (0.31–0.72)	0.88 (0.68–0.97)	0.33 (0.12–0.62)	0.68 (0.49–0.83)	0.62 (0.24–0.91)
Absolute lymphocyte count	1.21	0.42 (0.24–0.61)	0.17 (0.05–0.37)	0.93 (0.68–1.00)	0.80 (0.28–0.99)	0.41 (0.25–0.59)
Absolute neutrophil count	4.09	0.57 (0.37–0.76)	0.83 (0.63–0.95)	0.40 (0.16–0.68)	0.69 (0.49–0.85)	0.60 (0.26–0.88)
Absolute monocyte count	0.43	0.54 (0.36–0.73)	0.21 (0.07–0.42)	1.00 (0.78–1.00)	1.00 (0.48–1.00)	0.44 (0.27–0.62)
Absolute eosinophil count	0.25	0.51 (0.32–0.70)	0.29 (0.13–0.51)	0.87 (0.60–0.98)	0.78 (0.40–0.97)	0.43 (0.25–0.63)
Eosinophil	6.65	0.52 (0.33–0.71)	0.58 (0.37–0.78)	0.67 (0.38–0.88)	0.74 (0.49–0.91)	0.50 (0.27–0.73)
Eosinophil-to-lymphocyte ratio	0.21	0.54 (0.35–0.73)	0.54 (0.33–0.74)	0.73 (0.45–0.92)	0.76 (0.50–0.93)	0.50 (0.28–0.72)
Systemic inflammatory response index	0.95	0.64 (0.46–0.83)	0.58 (0.37–0.78)	0.80 (0.52–0.96)	0.82 (0.57–0.96)	0.55 (0.32–0.76)
Lactate dehydrogenase	179.00	0.55 (0.37–0.74)	0.65 (0.43–0.84)	0.53 (0.27–0.79)	0.68 (0.45–0.86)	0.50 (0.25–0.75)
Eosinophil cationic protein	23.20	0.45 (0.26–0.64)	0.54 (0.33–0.74)	0.53 (0.27–0.79)	0.65 (0.41–0.85)	0.42 (0.20–0.67)
Total serum IgE	110.90	0.52 (0.34–0.71)	0.38 (0.19–0.59)	0.87 (0.60–0.98)	0.82 (0.48–0.98)	0.46 (0.28–0.66)
Malassezia-specific IgE	0.07	0.51 (0.33–0.70)	0.25 (0.10–0.47)	1.00 (0.78–1.00)	1.00 (0.54–1.00)	0.45 (0.28–0.64)

III. Achievement of EASI-100 response (week 40)

Predictor	Cut-off	AUC (95% CI)	Sensitivity (95% CI)	Specificity (95% CI)	PPV (95% CI)	NPV (95% CI)
White blood cell count	6.04	0.55 (0.31–0.79)	0.56 (0.21–0.86)	0.67 (0.47–0.83)	0.33 (0.12–0.62)	0.83 (0.63–0.95)
Absolute lymphocyte count	1.56	0.47 (0.22–0.72)	0.44 (0.14–0.79)	0.70 (0.51–0.85)	0.31 (0.09–0.61)	0.81 (0.61–0.93)
Absolute neutrophil count	2.41	0.63 (0.37–0.89)	0.44 (0.14–0.79)	0.93 (0.78–0.99)	0.67 (0.22–0.96)	0.85 (0.68–0.95)
Absolute monocyte count	0.54	0.66 (0.42–0.89)	0.67 (0.30–0.93)	0.73 (0.54–0.88)	0.43 (0.18–0.71)	0.88 (0.69–0.97)
Absolute eosinophil count	0.32	0.59 (0.34–0.84)	0.56 (0.21–0.86)	0.70 (0.51–0.85)	0.36 (0.13–0.65)	0.84 (0.64–0.95)
Eosinophil	4.65	0.59 (0.33–0.84)	0.56 (0.21–0.86)	0.77 (0.58–0.90)	0.42 (0.15–0.72)	0.85 (0.66–0.96)
Eosinophil-to-lymphocyte ratio	0.21	0.60 (0.37–0.83)	0.67 (0.30–0.93)	0.63 (0.44–0.80)	0.35 (0.14–0.62)	0.86 (0.65–0.97)
Systemic inflammatory response index	0.76	0.69 (0.47–0.91)	0.56 (0.21–0.86)	0.83 (0.65–0.94)	0.50 (0.19–0.81)	0.86 (0.68–0.96)
Lactate dehydrogenase	174.50	0.57 (0.30–0.84)	0.56 (0.21–0.86)	0.72 (0.53–0.87)	0.38 (0.14–0.68)	0.84 (0.64–0.95)
Eosinophil cationic protein	17.80	0.55 (0.29–0.80)	0.56 (0.21–0.86)	0.70 (0.51–0.85)	0.36 (0.13–0.65)	0.84 (0.64–0.95)
Total serum IgE	110.90	0.52 (0.29–0.75)	0.44 (0.14–0.79)	0.77 (0.58–0.90)	0.36 (0.11–0.69)	0.82 (0.63–0.94)
Malassezia-specific IgE	0.55	0.53 (0.30–0.76)	0.78 (0.40–0.97)	0.40 (0.23–0.59)	0.28 (0.12–0.49)	0.86 (0.57–0.98)

IV. Achievement of EASI < 7 (week 40)

Predictor	Cut-off	AUC (95% CI)	Sensitivity (95% CI)	Specificity (95% CI)	PPV (95% CI)	NPV (95% CI)
White blood cell count	6.78	0.54 (0.31–0.76)	0.68 (0.48–0.84)	0.55 (0.23–0.83)	0.79 (0.58–0.93)	0.40 (0.16–0.68)
Absolute lymphocyte count	1.58	0.45 (0.26–0.64)	0.39 (0.22–0.59)	0.73 (0.39–0.94)	0.79 (0.49–0.95)	0.32 (0.15–0.54)
Absolute neutrophil count	4.09	0.59 (0.36–0.82)	0.82 (0.63–0.94)	0.45 (0.17–0.77)	0.79 (0.60–0.92)	0.50 (0.19–0.81)
Absolute monocyte count	0.54	0.55 (0.36–0.73)	0.43 (0.24–0.63)	0.82 (0.48–0.98)	0.86 (0.57–0.98)	0.36 (0.18–0.57)
Absolute eosinophil count	1.14	0.53 (0.33–0.72)	0.25 (0.11–0.45)	0.91 (0.59–1.00)	0.88 (0.47–1.00)	0.32 (0.17–0.51)
Eosinophil	6.65	0.46 (0.26–0.67)	0.54 (0.34–0.72)	0.64 (0.31–0.89)	0.79 (0.54–0.94)	0.35 (0.15–0.59)
Eosinophil-to-lymphocyte ratio	0.53	0.51 (0.31–0.72)	0.25 (0.11–0.45)	0.91 (0.59–1.00)	0.88 (0.47–1.00)	0.32 (0.17–0.51)
Systemic inflammatory response index	1.18	0.66 (0.47–0.85)	0.68 (0.48–0.84)	0.73 (0.39–0.94)	0.86 (0.65–0.97)	0.47 (0.23–0.72)
Lactate dehydrogenase	179.00	0.60 (0.41–0.79)	0.67 (0.46–0.83)	0.64 (0.31–0.89)	0.82 (0.60–0.95)	0.44 (0.20–0.70)
Eosinophil cationic protein	17.80	0.48 (0.28–0.69)	0.39 (0.22–0.59)	0.73 (0.39–0.94)	0.79 (0.49–0.95)	0.32 (0.15–0.54)
Total serum IgE	125.50	0.59 (0.38–0.79)	0.39 (0.22–0.59)	0.91 (0.59–1.00)	0.92 (0.62–1.00)	0.37 (0.19–0.58)
Malassezia-specific IgE	0.09	0.59 (0.39–0.79)	0.25 (0.11–0.45)	1.00 (0.72–1.00)	1.00 (0.59–1.00)	0.34 (0.19–0.53)

Notes: [a] Available for 39 patients (one missing value). All predictors are week 16 (on-treatment) values. Optimal cut-off determined by maximising the Youden index (sensitivity + specificity – 1). All analyses performed on complete cases only. Abbreviations: AUC, area under the receiver-operating characteristic curve; EASI, Eczema Area and Severity Index; ECP, eosinophilic cationic protein; ELR, eosinophil-to-lymphocyte ratio; LDH, lactate dehydrogenase; SIRI, systemic inflammatory response index.

Table S1b. Diagnostic performance of week 16 IgE class variables for predicting EASI response at week 40 (highest class considered as positive test result).

Variable	Outcome	N	Accuracy	Sensitivity	Specificity	PPV	NPV	AUC
**I. Achievement of EASI-75 response**								
Total serum IgE class	EASI-75 at Week 40	39	0.49	0.52	0.40	0.71	0.22	0.41
Malassezia-specific IgE class	EASI-75 at Week 40	39	0.41	0.38	0.50	0.69	0.22	0.44
**II. Achievement of EASI-90 response**								
Total serum IgE class	EASI-90 at Week 40	39	0.51	0.54	0.47	0.62	0.39	0.45
Malassezia-specific IgE class	EASI-90 at Week 40	39	0.54	0.46	0.67	0.69	0.43	0.56
**III. Achievement of EASI-100 response**								
Total serum IgE class	EASI-100 at Week 40	39	0.49	0.56	0.47	0.24	0.78	0.46
Malassezia-specific IgE class	EASI-100 at Week 40	39	0.56	0.44	0.60	0.25	0.78	0.52
**IV. Achievement of EASI score < 7 (low disease activity)**								
Total serum IgE class	EASI < 7 at Week 40	39	0.46	0.50	0.36	0.67	0.22	0.39
Malassezia-specific IgE class	EASI < 7 at Week 40	39	0.44	0.39	0.55	0.69	0.26	0.47

Notes: All predictors are week 16 (on-treatment) values. Positive test result was defined as the highest IgE class group (Class III–IV for total serum IgE; Class III–VI for Malassezia-specific IgE). Outcome positive class = "Yes" for all binary outcomes. AUC calculated by treating the IgE class as an ordinal numeric variable. All analyses performed on complete cases (N = 39; one patient lost to follow-up after week 16). Abbreviations: EASI, Eczema Area and Severity Index; NPV, negative predictive value; PPV, positive predictive value.

Table S2a *ROC discrimination of baseline continuous biomarkers at week 16, with 95% CIs.*

I Achievement of EASI-75, week 16

Predictor	Cut-off	AUC (95% CI)	Sensitivity (95% CI)	Specificity (95% CI)	PPV (95% CI)	NPV (95% CI)
White blood cell count	4.96	0.53 (0.31–0.75)	0.93 (0.77–0.99)	0.27 (0.06–0.61)	0.77 (0.60–0.90)	0.60 (0.15–0.95)
Absolute lymphocyte count	1.41	0.57 (0.35–0.80)	0.69 (0.49–0.85)	0.64 (0.31–0.89)	0.83 (0.63–0.95)	0.44 (0.20–0.70)
Absolute neutrophil count	2.85	0.59 (0.36–0.82)	0.86 (0.68–0.96)	0.45 (0.17–0.77)	0.81 (0.63–0.93)	0.56 (0.21–0.86)
Absolute monocyte count	0.46	0.50 (0.28–0.71)	0.90 (0.73–0.98)	0.27 (0.06–0.61)	0.76 (0.59–0.89)	0.50 (0.12–0.88)
Absolute eosinophil count	0.47	0.55 (0.34–0.76)	0.48 (0.29–0.67)	0.73 (0.39–0.94)	0.82 (0.57–0.96)	0.35 (0.16–0.57)
Eosinophil	6.45	0.52 (0.30–0.73)	0.59 (0.39–0.76)	0.55 (0.23–0.83)	0.77 (0.55–0.92)	0.33 (0.13–0.59)
Eosinophil-to-lymphocyte ratio	0.34	0.55 (0.35–0.76)	0.38 (0.21–0.58)	0.82 (0.48–0.98)	0.85 (0.55–0.98)	0.33 (0.17–0.54)
Systemic inflammatory response index	1.10	0.52 (0.30–0.75)	0.72 (0.53–0.87)	0.45 (0.17–0.77)	0.78 (0.58–0.91)	0.38 (0.14–0.68)
Lactate dehydrogenase	283.50	0.60 (0.42–0.79)	0.36 (0.19–0.56)	0.91 (0.59–1.00)	0.91 (0.59–1.00)	0.36 (0.19–0.56)
Eosinophil cationic protein	9.74	0.50 (0.30–0.71)	0.14 (0.04–0.32)	1.00 (0.72–1.00)	1.00 (0.40–1.00)	0.31 (0.16–0.48)

Predictor	Cut-off	AUC (95% CI)	Sensitivity (95% CI)	Specificity (95% CI)	PPV (95% CI)	NPV (95% CI)
Total serum IgE	3,111.50	0.56 (0.36–0.75)	0.52 (0.33–0.71)	0.64 (0.31–0.89)	0.79 (0.54–0.94)	0.33 (0.15–0.57)
Malassezia-specific IgE	0.10	0.52 (0.31–0.73)	0.21 (0.08–0.40)	1.00 (0.72–1.00)	1.00 (0.54–1.00)	0.32 (0.17–0.51)

II Achievement of EASI-90, week 16

Predictor	Cut-off	AUC (95% CI)	Sensitivity (95% CI)	Specificity (95% CI)	PPV (95% CI)	NPV (95% CI)
White blood cell count	6.89	0.53 (0.34–0.72)	0.79 (0.49–0.95)	0.50 (0.30–0.70)	0.46 (0.26–0.67)	0.81 (0.54–0.96)
Absolute lymphocyte count	1.00	0.46 (0.25–0.66)	0.21 (0.05–0.51)	0.92 (0.75–0.99)	0.60 (0.15–0.95)	0.69 (0.51–0.83)
Absolute neutrophil count	4.20	0.48 (0.29–0.67)	0.79 (0.49–0.95)	0.42 (0.23–0.63)	0.42 (0.23–0.63)	0.79 (0.49–0.95)
Absolute monocyte count	0.58	0.55 (0.34–0.76)	0.64 (0.35–0.87)	0.58 (0.37–0.77)	0.45 (0.23–0.68)	0.75 (0.51–0.91)
Absolute eosinophil count	0.39	0.56 (0.36–0.75)	0.71 (0.42–0.92)	0.50 (0.30–0.70)	0.43 (0.23–0.66)	0.76 (0.50–0.93)
Eosinophil	6.10	0.56 (0.36–0.75)	0.71 (0.42–0.92)	0.46 (0.27–0.67)	0.42 (0.22–0.63)	0.75 (0.48–0.93)
Eosinophil-to-lymphocyte ratio	0.50	0.58 (0.37–0.78)	0.36 (0.13–0.65)	0.88 (0.70–0.98)	0.62 (0.24–0.91)	0.72 (0.53–0.86)
Systemic inflammatory response index	2.49	0.49 (0.29–0.69)	0.21 (0.05–0.51)	0.88 (0.70–0.98)	0.50 (0.12–0.88)	0.68 (0.49–0.83)
Lactate dehydrogenase	206.00	0.63 (0.42–0.83)	0.43 (0.18–0.71)	0.88 (0.69–0.97)	0.67 (0.30–0.93)	0.73 (0.54–0.88)
Eosinophil cationic protein	28.20	0.60 (0.39–0.80)	0.50 (0.23–0.77)	0.77 (0.56–0.91)	0.54 (0.25–0.81)	0.74 (0.54–0.89)
Total serum IgE	259.00	0.54 (0.34–0.73)	0.43 (0.18–0.71)	0.81 (0.61–0.93)	0.55 (0.23–0.83)	0.72 (0.53–0.87)
Malassezia-specific IgE	5.14	0.58 (0.38–0.78)	0.71 (0.42–0.92)	0.50 (0.30–0.70)	0.43 (0.23–0.66)	0.76 (0.50–0.93)

III Achievement of EASI < 7, week 16

Predictor	Cut-off	AUC (95% CI)	Sensitivity (95% CI)	Specificity (95% CI)	PPV (95% CI)	NPV (95% CI)
White blood cell count	6.89	0.67 (0.47–0.87)	0.71 (0.51–0.87)	0.67 (0.35–0.90)	0.83 (0.63–0.95)	0.50 (0.25–0.75)
Absolute lymphocyte count	2.19	0.58 (0.37–0.80)	0.93 (0.76–0.99)	0.33 (0.10–0.65)	0.76 (0.59–0.89)	0.67 (0.22–0.96)
Absolute neutrophil count	4.40	0.56 (0.34–0.78)	0.82 (0.63–0.94)	0.50 (0.21–0.79)	0.79 (0.60–0.92)	0.55 (0.23–0.83)
Absolute monocyte count	0.65	0.66 (0.48–0.84)	0.75 (0.55–0.89)	0.58 (0.28–0.85)	0.81 (0.61–0.93)	0.50 (0.23–0.77)
Absolute eosinophil count	0.47	0.52 (0.32–0.71)	0.46 (0.28–0.66)	0.67 (0.35–0.90)	0.76 (0.50–0.93)	0.35 (0.16–0.57)
Eosinophil	6.10	0.55 (0.35–0.74)	0.64 (0.44–0.81)	0.50 (0.21–0.79)	0.75 (0.53–0.90)	0.38 (0.15–0.65)
Eosinophil-to-lymphocyte ratio	0.34	0.61 (0.42–0.80)	0.43 (0.24–0.63)	0.92 (0.62–1.00)	0.92 (0.64–1.00)	0.41 (0.22–0.61)
Systemic inflammatory response index	1.81	0.54 (0.33–0.75)	0.82 (0.63–0.94)	0.42 (0.15–0.72)	0.77 (0.58–0.90)	0.50 (0.19–0.81)
Lactate dehydrogenase	221.50	0.58 (0.39–0.76)	0.44 (0.25–0.65)	0.83 (0.52–0.98)	0.86 (0.57–0.98)	0.40 (0.21–0.61)
Eosinophil cationic protein	20.20	0.61 (0.42–0.80)	0.64 (0.44–0.81)	0.75 (0.43–0.95)	0.86 (0.64–0.97)	0.47 (0.24–0.71)

Total serum IgE	259.00	0.67 (0.51–0.83)	0.39 (0.22–0.59)	1.00 (0.74–1.00)	1.00 (0.72–1.00)	0.41 (0.24–0.61)
Malassezia-specific IgE	10.20	0.62 (0.44–0.81)	0.75 (0.55–0.89)	0.50 (0.21–0.79)	0.78 (0.58–0.91)	0.46 (0.19–0.75)

Notes: AUC 95% CI by the DeLong method (continuous) or stratified bootstrap (ordinal classes; sensitivity, specificity, PPV and NPV with exact binomial (Clopper–Pearson) 95% intervals at the Youden operating point. Youden cut-offs are optimised in-sample and are exploratory. Complete-case analysis.

Table S2b. *ROC discrimination of baseline IgE class variables at week 16, with 95% CIs.*

I Achievement of EASI-75, week 16

Predictor	Cut-off	AUC (95% CI)	Sensitivity (95% CI)	Specificity (95% CI)	PPV (95% CI)	NPV (95% CI)
Total serum IgE class	highest class	0.47 (0.31–0.66)	0.59 (0.39–0.76)	0.36 (0.11–0.69)	0.71 (0.49–0.87)	0.25 (0.07–0.52)
Malassezia-specific IgE class	highest class	0.50 (0.33–0.68)	0.52 (0.33–0.71)	0.45 (0.17–0.77)	0.71 (0.48–0.89)	0.26 (0.09–0.51)

II Achievement of EASI-90, week 16

Predictor	Cut-off	AUC (95% CI)	Sensitivity (95% CI)	Specificity (95% CI)	PPV (95% CI)	NPV (95% CI)
Total serum IgE class	highest class	0.48 (0.30–0.64)	0.57 (0.29–0.82)	0.38 (0.20–0.59)	0.33 (0.16–0.55)	0.62 (0.35–0.85)
Malassezia-specific IgE class	highest class	0.56 (0.40–0.74)	0.43 (0.18–0.71)	0.42 (0.23–0.63)	0.29 (0.11–0.52)	0.58 (0.33–0.80)

III Achievement of EASI < 7, week 16

Predictor	Cut-off	AUC (95% CI)	Sensitivity (95% CI)	Specificity (95% CI)	PPV (95% CI)	NPV (95% CI)
Total serum IgE class	highest class	0.36 (0.22–0.50)	0.54 (0.34–0.72)	0.25 (0.05–0.57)	0.62 (0.41–0.81)	0.19 (0.04–0.46)
Malassezia-specific IgE class	highest class	0.58 (0.42–0.74)	0.46 (0.28–0.66)	0.33 (0.10–0.65)	0.62 (0.38–0.82)	0.21 (0.06–0.46)

Notes: AUC 95% CI by the DeLong method (continuous) or stratified bootstrap (ordinal classes); sensitivity, specificity, PPV and NPV with exact binomial (Clopper–Pearson) 95% intervals at the Youden operating point. Youden cut-offs are optimised in-sample and are exploratory. Complete-case analysis.

Table S3a. *ROC discrimination of baseline continuous biomarkers at week 40, with 95% CIs.*

I Achievement of EASI-75, week 40

Predictor	Cut-off	AUC (95% CI)	Sensitivity (95% CI)	Specificity (95% CI)	PPV (95% CI)	NPV (95% CI)
White blood cell count	6.03	0.58 (0.36–0.79)	0.38 (0.21–0.58)	0.80 (0.44–0.97)	0.85 (0.55–0.98)	0.31 (0.14–0.52)
Absolute lymphocyte count	1.85	0.59 (0.40–0.78)	0.38 (0.21–0.58)	0.90 (0.55–1.00)	0.92 (0.62–1.00)	0.33 (0.17–0.54)
Absolute neutrophil count	4.67	0.60 (0.38–0.82)	0.86 (0.68–0.96)	0.40 (0.12–0.74)	0.81 (0.63–0.93)	0.50 (0.16–0.84)
Absolute monocyte count	0.71	0.51 (0.31–0.70)	0.28 (0.13–0.47)	0.90 (0.55–1.00)	0.89 (0.52–1.00)	0.30 (0.15–0.49)
Absolute eosinophil count	0.47	0.51 (0.28–0.74)	0.66 (0.46–0.82)	0.60 (0.26–0.88)	0.83 (0.61–0.95)	0.38 (0.15–0.65)
Eosinophil	7.45	0.50 (0.27–0.72)	0.66 (0.46–0.82)	0.50 (0.19–0.81)	0.79 (0.58–0.93)	0.33 (0.12–0.62)

Eosinophil-to-lymphocyte ratio	0.33	0.49 (0.26–0.72)	0.69 (0.49–0.85)	0.50 (0.19–0.81)	0.80 (0.59–0.93)	0.36 (0.13–0.65)
Systemic inflammatory response index	1.94	0.63 (0.40–0.85)	0.86 (0.68–0.96)	0.50 (0.19–0.81)	0.83 (0.65–0.94)	0.56 (0.21–0.86)
Lactate dehydrogenase	240.00	0.66 (0.47–0.85)	0.68 (0.48–0.84)	0.70 (0.35–0.93)	0.86 (0.65–0.97)	0.44 (0.20–0.70)
Eosinophil cationic protein	18.55	0.48 (0.24–0.71)	0.55 (0.36–0.74)	0.60 (0.26–0.88)	0.80 (0.56–0.94)	0.32 (0.13–0.57)
Total serum IgE	324.50	0.58 (0.38–0.78)	0.38 (0.21–0.58)	0.90 (0.55–1.00)	0.92 (0.62–1.00)	0.33 (0.17–0.54)
Malassezia-specific IgE	0.33	0.64 (0.45–0.82)	0.38 (0.21–0.58)	1.00 (0.69–1.00)	1.00 (0.72–1.00)	0.36 (0.19–0.56)

II Achievement of EASI-90, week 40

Predictor	Cut-off	AUC (95% CI)	Sensitivity (95% CI)	Specificity (95% CI)	PPV (95% CI)	NPV (95% CI)
White blood cell count	7.71	0.47 (0.28–0.67)	0.83 (0.63–0.95)	0.27 (0.08–0.55)	0.65 (0.45–0.81)	0.50 (0.16–0.84)
Absolute lymphocyte count	1.85	0.62 (0.44–0.79)	0.42 (0.22–0.63)	0.87 (0.60–0.98)	0.83 (0.52–0.98)	0.48 (0.29–0.68)
Absolute neutrophil count	4.67	0.45 (0.25–0.65)	0.83 (0.63–0.95)	0.27 (0.08–0.55)	0.65 (0.45–0.81)	0.50 (0.16–0.84)
Absolute monocyte count	0.59	0.57 (0.38–0.75)	0.62 (0.41–0.81)	0.60 (0.32–0.84)	0.71 (0.48–0.89)	0.50 (0.26–0.74)
Absolute eosinophil count	0.47	0.57 (0.37–0.77)	0.71 (0.49–0.87)	0.60 (0.32–0.84)	0.74 (0.52–0.90)	0.56 (0.30–0.80)
Eosinophil	6.90	0.60 (0.41–0.79)	0.62 (0.41–0.81)	0.67 (0.38–0.88)	0.75 (0.51–0.91)	0.53 (0.29–0.76)
Eosinophil-to-lymphocyte ratio	0.18	0.58 (0.39–0.77)	0.38 (0.19–0.59)	0.87 (0.60–0.98)	0.82 (0.48–0.98)	0.46 (0.28–0.66)
Systemic inflammatory response index	1.31	0.59 (0.41–0.78)	0.62 (0.41–0.81)	0.67 (0.38–0.88)	0.75 (0.51–0.91)	0.53 (0.29–0.76)
Lactate dehydrogenase	240.00	0.57 (0.38–0.76)	0.65 (0.43–0.84)	0.53 (0.27–0.79)	0.68 (0.45–0.86)	0.50 (0.25–0.75)
Eosinophil cationic protein	14.50	0.52 (0.33–0.72)	0.42 (0.22–0.63)	0.80 (0.52–0.96)	0.77 (0.46–0.95)	0.46 (0.27–0.67)
Total serum IgE	324.50	0.53 (0.35–0.71)	0.38 (0.19–0.59)	0.80 (0.52–0.96)	0.75 (0.43–0.95)	0.44 (0.25–0.65)
Malassezia-specific IgE	10.20	0.50 (0.31–0.69)	0.38 (0.19–0.59)	0.80 (0.52–0.96)	0.75 (0.43–0.95)	0.44 (0.25–0.65)

III Achievement of EASI-100, week 40

Predictor	Cut-off	AUC (95% CI)	Sensitivity (95% CI)	Specificity (95% CI)	PPV (95% CI)	NPV (95% CI)
White blood cell count	6.03	0.51 (0.30–0.72)	0.44 (0.14–0.79)	0.70 (0.51–0.85)	0.31 (0.09–0.61)	0.81 (0.61–0.93)
Absolute lymphocyte count	1.85	0.54 (0.31–0.78)	0.44 (0.14–0.79)	0.73 (0.54–0.88)	0.33 (0.10–0.65)	0.81 (0.62–0.94)
Absolute neutrophil count	5.04	0.45 (0.26–0.64)	1.00 (0.66–1.00)	0.17 (0.06–0.35)	0.26 (0.13–0.44)	1.00 (0.48–1.00)
Absolute monocyte count	0.48	0.59 (0.33–0.86)	0.44 (0.14–0.79)	0.87 (0.69–0.96)	0.50 (0.16–0.84)	0.84 (0.66–0.95)
Absolute eosinophil count	0.18	0.59 (0.38–0.81)	0.33 (0.07–0.70)	0.90 (0.73–0.98)	0.50 (0.12–0.88)	0.82 (0.65–0.93)
Eosinophil	9.45	0.59 (0.36–0.81)	1.00 (0.66–1.00)	0.27 (0.12–0.46)	0.29 (0.14–0.48)	1.00 (0.63–1.00)
Eosinophil-to-lymphocyte ratio	0.52	0.61 (0.40–0.82)	1.00 (0.66–1.00)	0.23 (0.10–0.42)	0.28 (0.14–0.47)	1.00 (0.59–1.00)

Predictor	Cut-off	AUC (95% CI)	Sensitivity (95% CI)	Specificity (95% CI)	PPV (95% CI)	NPV (95% CI)
Systemic inflammatory response index	1.21	0.60 (0.37–0.82)	0.67 (0.30–0.93)	0.67 (0.47–0.83)	0.38 (0.15–0.65)	0.87 (0.66–0.97)
Lactate dehydrogenase	240.00	0.59 (0.35–0.82)	0.78 (0.40–0.97)	0.48 (0.29–0.67)	0.32 (0.14–0.55)	0.88 (0.62–0.98)
Eosinophil cationic protein	30.20	0.54 (0.33–0.75)	0.89 (0.52–1.00)	0.30 (0.15–0.49)	0.28 (0.13–0.47)	0.90 (0.55–1.00)
Total serum IgE	324.50	0.54 (0.33–0.76)	0.44 (0.14–0.79)	0.73 (0.54–0.88)	0.33 (0.10–0.65)	0.81 (0.62–0.94)
Malassezia-specific IgE	3.38	0.53 (0.31–0.74)	0.67 (0.30–0.93)	0.53 (0.34–0.72)	0.30 (0.12–0.54)	0.84 (0.60–0.97)

IV Achievement of EASI < 7, week 40

Predictor	Cut-off	AUC (95% CI)	Sensitivity (95% CI)	Specificity (95% CI)	PPV (95% CI)	NPV (95% CI)
White blood cell count	7.80	0.52 (0.30–0.73)	0.86 (0.67–0.96)	0.27 (0.06–0.61)	0.75 (0.57–0.89)	0.43 (0.10–0.82)
Absolute lymphocyte count	1.85	0.62 (0.43–0.81)	0.39 (0.22–0.59)	0.91 (0.59–1.00)	0.92 (0.62–1.00)	0.37 (0.19–0.58)
Absolute neutrophil count	4.67	0.53 (0.30–0.76)	0.86 (0.67–0.96)	0.36 (0.11–0.69)	0.77 (0.59–0.90)	0.50 (0.16–0.84)
Absolute monocyte count	0.45	0.55 (0.35–0.74)	0.18 (0.06–0.37)	1.00 (0.72–1.00)	1.00 (0.48–1.00)	0.32 (0.17–0.51)
Absolute eosinophil count	0.47	0.50 (0.28–0.71)	0.64 (0.44–0.81)	0.55 (0.23–0.83)	0.78 (0.56–0.93)	0.38 (0.15–0.65)
Eosinophil	6.90	0.51 (0.30–0.72)	0.57 (0.37–0.76)	0.64 (0.31–0.89)	0.80 (0.56–0.94)	0.37 (0.16–0.62)
Eosinophil-to-lymphocyte ratio	0.27	0.50 (0.29–0.72)	0.61 (0.41–0.78)	0.55 (0.23–0.83)	0.77 (0.55–0.92)	0.35 (0.14–0.62)
Systemic inflammatory response index	1.31	0.64 (0.43–0.85)	0.61 (0.41–0.78)	0.73 (0.39–0.94)	0.85 (0.62–0.97)	0.42 (0.20–0.67)
Lactate dehydrogenase	221.50	0.64 (0.45–0.82)	0.48 (0.29–0.68)	0.91 (0.59–1.00)	0.93 (0.66–1.00)	0.42 (0.22–0.63)
Eosinophil cationic protein	18.55	0.49 (0.27–0.71)	0.57 (0.37–0.76)	0.64 (0.31–0.89)	0.80 (0.56–0.94)	0.37 (0.16–0.62)
Total serum IgE	324.50	0.58 (0.39–0.77)	0.39 (0.22–0.59)	0.91 (0.59–1.00)	0.92 (0.62–1.00)	0.37 (0.19–0.58)
Malassezia-specific IgE	0.33	0.59 (0.40–0.78)	0.36 (0.19–0.56)	0.91 (0.59–1.00)	0.91 (0.59–1.00)	0.36 (0.19–0.56)

Notes: AUC 95% CI by the DeLong method (continuous) or stratified bootstrap (ordinal classes) [4,5]; sensitivity, specificity, PPV and NPV with exact binomial (Clopper–Pearson) 95% intervals at the Youden operating point [6]. Youden cut-offs are optimised in-sample and are exploratory [7]. Complete-case analysis.

Table S3b. *ROC discrimination of baseline IgE class variables at week 40, with 95% CIs.*

I Achievement of EASI-75, week 40

Predictor	Cut-off	AUC (95% CI)	Sensitivity (95% CI)	Specificity (95% CI)	PPV (95% CI)	NPV (95% CI)
Total serum IgE class	highest class	0.41 (0.26–0.59)	0.55 (0.36–0.74)	0.30 (0.07–0.65)	0.70 (0.47–0.87)	0.19 (0.04–0.46)
Malassezia-specific IgE class	highest class	0.58 (0.39–0.76)	0.48 (0.29–0.67)	0.40 (0.12–0.74)	0.70 (0.46–0.88)	0.21 (0.06–0.46)

II Achievement of EASI-90, week 40

Predictor	Cut-off	AUC (95% CI)	Sensitivity (95% CI)	Specificity (95% CI)	PPV (95% CI)	NPV (95% CI)
Total serum IgE class	highest class	0.45 (0.29–0.62)	0.54 (0.33–0.74)	0.33 (0.12–0.62)	0.57 (0.34–0.77)	0.31 (0.11–0.59)
Malassezia-specific IgE class	highest class	0.52 (0.35–0.69)	0.54 (0.33–0.74)	0.53 (0.27–0.79)	0.65 (0.41–0.85)	0.42 (0.20–0.67)

III Achievement of EASI-100, week 40

Predictor	Cut-off	AUC (95% CI)	Sensitivity (95% CI)	Specificity (95% CI)	PPV (95% CI)	NPV (95% CI)
Total serum IgE class	highest class	0.48 (0.30–0.66)	0.56 (0.21–0.86)	0.40 (0.23–0.59)	0.22 (0.07–0.44)	0.75 (0.48–0.93)
Malassezia-specific IgE class	highest class	0.59 (0.41–0.76)	0.67 (0.30–0.93)	0.53 (0.34–0.72)	0.30 (0.12–0.54)	0.84 (0.60–0.97)

IV Achievement of EASI < 7, week 40

Predictor	Cut-off	AUC (95% CI)	Sensitivity (95% CI)	Specificity (95% CI)	PPV (95% CI)	NPV (95% CI)
Total serum IgE class	highest class	0.39 (0.24–0.55)	0.54 (0.34–0.72)	0.27 (0.06–0.61)	0.65 (0.43–0.84)	0.19 (0.04–0.46)
Malassezia-specific IgE class	highest class	0.55 (0.36–0.72)	0.50 (0.31–0.69)	0.45 (0.17–0.77)	0.70 (0.46–0.88)	0.26 (0.09–0.51)

Notes: AUC 95% CI by the DeLong method (continuous) or stratified bootstrap (ordinal classes); sensitivity, specificity, PPV and NPV with exact binomial (Clopper–Pearson) 95% intervals at the Youden operating point. Youden cut-offs are optimised in-sample and are exploratory. Complete-case analysis.

Univariate Logistic Regression Analysis

To further delineate the independent predictive value of biomarkers identified as noteworthy in the ROC analysis, univariate logistic regression models were constructed for each candidate predictor, yielding crude odds ratios (OR) with 95% confidence intervals. The number of covariates in any single model was restricted to approximately 10 events per variable to mitigate overfitting. Model calibration was assessed using the Hosmer–Lemeshow goodness-of-fit test, and the Nagelkerke pseudo-R^2 was reported as an indicator of the proportion of outcome variance explained.

Tables below present the results of univariate logistic regression models evaluating baseline laboratory biomarkers as predictors of clinical response to dupilumab. Each predictor was entered individually. For total serum IgE, the pre-specified ordinal class is the primary predictor; the continuous total-IgE model (rows labelled “Total serum IgE (kU/L)”) is reported only as a sensitivity analysis, owing to the interval censoring, and is not the primary total-IgE inference. Categorical IgE class variables were coded as binary (highest severity class = 1, reference = 0). For total serum IgE class, the positive class was defined as class III–IV versus 0/I–II. For Malassezia-specific IgE class, the positive class was defined as class III–VI versus 0–II.

Table S4a. Univariate logistic regression: baseline predictors of EASI-75 (Week 16).

Predictor	n	OR	95% CI	p-value	Nagelkerke R^2	p (H–L)
White blood cell count (×10³/µL)	40	1.12	0.74–1.93	0.460	0.02	0.347
Absolute lymphocyte count (×10³/µL)	40	1.24	0.38–4.05	0.722	< 0.01	0.179
Absolute neutrophil count (×10³/µL)	40	1.25	0.66–2.36	0.487	0.02	0.684
Systemic inflammatory response index (SIRI)	40	0.97	0.39–2.40	0.950	< 0.01	0.886
Absolute monocyte count (×10³/µL)	40	0.71	0.01–35.65	0.863	< 0.01	0.387
Absolute eosinophil count (×10³/µL)	40	2.31	0.19–27.87	0.510	0.02	0.590
Eosinophil-to-lymphocyte ratio (ELR)	40	3.09	0.07–146.55	0.566	0.01	0.905
Eosinophil (%)	40	1.01	0.84–1.22	0.889	< 0.01	0.874
Lactate dehydrogenase (LDH, U/L)	39	1.01	0.99–1.02	0.332	0.04	0.502
Eosinophil cationic protein (ECP, µg/L)	40	1.00	0.95–1.05	0.990	< 0.01	0.193
Total serum IgE (kU/L)) *[sensitivity]*	40	1.00	1.00–1.00	0.600	0.01	0.999
Malassezia-specific IgE (kUA/L)	40	1.01	0.97–1.04	0.751	< 0.01	0.635
Total serum IgE class (III–IV vs. 0/I–II)	40	0.81	0.19–3.34	0.773	< 0.01	–
Malassezia-specific IgE class (III–VI vs. 0–II)	40	0.89	0.22–3.59	0.873	< 0.01	–

Notes: ECP, eosinophil cationic protein; ELR, Eosinophil-to-lymphocyte ratio; OR, odds ratio; 95% CI, 95% Wald confidence interval for the OR; LDH, Lactate dehydrogenase; Nagelkerke R^2, Nagelkerke pseudo-R-squared; p (H–L), p-value from the Hosmer–Lemeshow goodness-of-fit test; SIRI, systemic inflammatory response index.

Table S4b. Univariate logistic regression: baseline predictors of EASI-90 (Week 16).

Predictor	n	OR	95% CI	p-value	Nagelkerke R^2	p (H–L)
White blood cell count (×10³/µL)	40	1.19	0.81–1.74	0.372	0.03	0.072
Absolute lymphocyte count (×10³/µL)	40	1.49	0.52–4.30	0.462	0.03	**0.044**
Absolute neutrophil count (×10³/µL)	40	1.12	0.65–1.93	0.677	0.01	0.326
Systemic inflammatory response index (SIRI)	40	1.10	0.48–2.56	0.817	< 0.01	0.780
Absolute monocyte count (×10³/µL)	40	3.06	0.08–120.30	0.550	0.01	0.084
Absolute eosinophil count (×10³/µL)	40	4.47	0.51–39.24	0.176	0.07	0.450
Eosinophil-to-lymphocyte ratio (ELR)	40	8.43	0.27–263.69	0.225	0.05	0.701
Eosinophil (%)	40	1.10	0.92–1.32	0.309	0.04	0.800
Lactate dehydrogenase (LDH, U/L)	39	0.99	0.98–1.01	0.303	0.04	0.444
Eosinophil cationic protein (ECP, µg/L)	40	1.04	0.99–1.09	0.160	0.07	0.960
Total serum IgE (kU/L)) *[sensitivity]*	40	1.00	1.000–1.000	0.629	0.01	0.343
Malassezia-specific IgE (kUA/L)	40	1.00	0.970–1.028	0.927	< 0.01	0.782
Total serum IgE class (III–IV vs. 0/I–II)	40	0.83	0.222–3.122	0.787	< 0.01	–
Malassezia-specific IgE class (III–VI vs. 0–II)	40	0.55	0.148–2.046	0.372	0.03	–

Notes: ECP, eosinophil cationic protein; ELR, Eosinophil-to-lymphocyte ratio; OR, odds ratio; 95% CI, 95% Wald confidence interval for the OR; LDH, Lactate dehydrogenase; Nagelkerke R^2, Nagelkerke pseudo-R-squared; p (H–L), p-value from the Hosmer–Lemeshow goodness-of-fit test; SIRI, systemic inflammatory response index. Bold p-values indicate statistical significance at $\alpha = 0.05$.

Table S4c. Univariate logistic regression: baseline predictors of EASI < 7 (Week 16).

Predictor	n	OR	95% CI	p-value	Nagelkerke R^2	p (H–L)
White blood cell count (×10³/µL)	40	0.90	0.62–1.32	0.599	0.01	0.219
Absolute lymphocyte count (×10³/µL)	40	0.63	0.21–1.87	0.400	0.02	0.403
Absolute neutrophil count (×10³/µL)	40	0.91	0.52–1.60	0.746	< 0.01	0.351
Systemic inflammatory response index (SIRI)	40	0.81	0.34–1.91	0.627	0.01	0.846
Absolute monocyte count (×10³/µL)	40	0.07	0.001–3.36	0.178	0.07	0.529
Absolute eosinophil count (×10³/µL)	40	1.96	0.19–20.34	0.573	0.01	0.808
Eosinophil-to-lymphocyte ratio (ELR)	40	11.02	0.18–664.07	0.251	0.05	0.657
Eosinophil (%)	40	1.07	0.88–1.30	0.486	0.02	0.601
Lactate dehydrogenase (LDH, U/L)	39	1.00	0.99–1.01	0.594	0.01	0.442
Eosinophil cationic protein (ECP, µg/L)	40	0.98	0.94–1.03	0.434	0.02	0.098
Total serum IgE (kU/L)) *[sensitivity]*	40	1.00	0.999–1.00	0.114	0.09	0.656
Malassezia-specific IgE (kUA/L)	40	1.00	0.97–1.03	0.833	< 0.01	0.862
Total serum IgE class (III–IV vs. 0/I–II)	40	0.39	0.09–1.73	0.213	0.06	–
Malassezia-specific IgE class (III–VI vs. 0–II)	40	0.43	0.11–1.778	0.246	0.05	–

Notes: ECP, eosinophil cationic protein; ELR, Eosinophil-to-lymphocyte ratio; OR, odds ratio; 95% CI, 95% Wald confidence interval for the OR; LDH, Lactate dehydrogenase; Nagelkerke R^2, Nagelkerke pseudo-R-squared; p (H–L), p-value from the Hosmer–Lemeshow goodness-of-fit test; SIRI, systemic inflammatory response index.

Table S4d. Univariate logistic regression: baseline predictors of EASI-75 (Week 40).

Predictor	n	OR	95% CI	p-value	Nagelkerke R^2	p (H–L)
White blood cell count (×10³/µL)	39	0.96	0.64–1.44	0.841	< 0.01	0.729
Absolute lymphocyte count (×10³/µL)	39	2.06	0.52–8.12	0.302	0.04	0.501
Absolute neutrophil count (×10³/µL)	39	0.75	0.42–1.33	0.320	0.04	0.069
Systemic inflammatory response index (SIRI)	39	0.55	0.23–1.35	0.192	0.06	0.150
Absolute monocyte count (×10³/µL)	39	2.31	0.03–159.44	0.698	0.01	0.789
Absolute eosinophil count (×10³/µL)	39	1.72	0.15–19.83	0.664	0.01	0.502
Eosinophil-to-lymphocyte ratio (ELR)	39	1.06	0.03–44.73	0.977	< 0.01	0.226
Eosinophil (%)	39	1.04	0.86–1.27	0.678	0.01	0.464
Lactate dehydrogenase (LDH, U/L)	38	0.99	0.98–1.00	0.194	0.07	0.394
Eosinophil cationic protein (ECP, µg/L)	39	1.01	0.95–1.06	0.820	< 0.01	0.477
Total serum IgE (kU/L)) *[sensitivity]*	39	1.00	0.999–1.00	0.364	0.03	0.141
Malassezia-specific IgE (kUA/L)	39	0.98	0.95–1.02	0.276	0.05	**0.048**
Total serum IgE class (III–IV vs. 0/I–II)	39	0.53	0.11–2.46	0.415	0.03	–
Malassezia-specific IgE class (III–VI vs. 0–II)	39	0.62	0.15–2.68	0.524	0.01	–

Notes: ECP, eosinophil cationic protein; ELR, Eosinophil-to-lymphocyte ratio; OR, odds ratio; 95% CI, 95% Wald confidence interval for the OR; LDH, Lactate dehydrogenase; Nagelkerke R^2, Nagelkerke pseudo-R-squared; p (H–L), p-value from the Hosmer–Lemeshow goodness-of-fit test; SIRI, systemic inflammatory response index. Bold p-values indicate statistical significance at $\alpha = 0.05$.

Table S4e. Univariate logistic regression: baseline predictors of EASI-90 (Week 40).

Predictor	n	OR	95% CI	p-value	Nagelkerke R^2	p (H–L)
White blood cell count (×10³/μL)	39	1.15	0.76–1.72	0.511	0.02	0.374
Absolute lymphocyte count (×10³/μL)	39	2.34	0.68–8.00	0.176	0.07	0.784
Absolute neutrophil count (×10³/μL)	39	1.09	0.63–1.88	0.757	< 0.01	0.690
Systemic inflammatory response index (SIRI)	39	0.74	0.32–1.70	0.480	0.01	0.122
Absolute monocyte count (×10³/μL)	39	0.58	0.02–21.71	0.769	< 0.01	0.063
Absolute eosinophil count (×10³/μL)	39	0.90	0.12–6.58	0.913	< 0.01	0.085
Eosinophil-to-lymphocyte ratio (ELR)	39	0.33	0.01–9.34	0.517	0.01	0.304
Eosinophil (%)	39	0.93	0.79–1.11	0.441	0.02	0.201
Lactate dehydrogenase (LDH, U/L)	38	0.99	0.98–1.01	0.349	0.03	0.622
Eosinophil cationic protein (ECP, μg/L)	39	1.00	0.95–1.05	0.986	< 0.01	0.697
Total serum IgE (kU/L)) *[sensitivity]*	39	1.00	1.00–1.00	0.615	0.01	0.411
Malassezia-specific IgE (kUA/L)	39	1.00	0.96–1.03	0.835	< 0.01	**0.030**
Total serum IgE class (III–IV vs. 0/I–II)	39	0.59	0.16–2.26	0.442	0.02	–
Malassezia-specific IgE class (III–VI vs. 0–II)	39	1.35	0.37–4.93	0.649	0.01	–

Notes: ECP, eosinophil cationic protein; ELR, Eosinophil-to-lymphocyte ratio; OR, odds ratio; 95% CI, 95% Wald confidence interval for the OR; LDH, Lactate dehydrogenase; Nagelkerke R^2, Nagelkerke pseudo-R-squared; p (H–L), p-value from the Hosmer–Lemeshow goodness-of-fit test; SIRI, systemic inflammatory response index. Bold p-values indicate statistical significance at $\alpha = 0.05$.

Table S4f. Univariate logistic regression: baseline predictors of EASI-100 (Week 40).

Predictor	n	OR	95% CI	p-value	Nagelkerke R^2	p (H–L)
White blood cell count (×10³/μL)	39	0.93	0.59–1.47	0.755	< 0.01	0.567
Absolute lymphocyte count (×10³/μL)	39	1.10	0.33–3.63	0.875	< 0.01	0.995
Absolute neutrophil count (×10³/μL)	39	0.97	0.52–1.82	0.929	< 0.01	0.141
Systemic inflammatory response index (SIRI)	39	0.63	0.20–1.96	0.421	0.03	0.135
Absolute monocyte count (×10³/μL)	39	0.13	0.001–15.40	0.400	0.03	0.401
Absolute eosinophil count (×10³/μL)	39	0.19	0.01–4.48	0.302	0.05	0.935
Eosinophil-to-lymphocyte ratio (ELR)	39	0.08	0.001–8.70	0.295	0.05	0.926
Eosinophil (%)	39	0.87	0.69–1.10	0.247	0.06	0.126
Lactate dehydrogenase (LDH, U/L)	38	0.99	0.98–1.01	0.222	0.06	0.266
Eosinophil cationic protein (ECP, μg/L)	39	0.99	0.93–1.05	0.611	0.01	0.566
Total serum IgE (kU/L)) *[sensitivity]*	39	1.00	1.00–1.00	0.613	0.01	0.581
Malassezia-specific IgE (kUA/L)	39	0.99	0.95–1.04	0.781	< 0.01	0.126
Total serum IgE class (III–IV vs. 0/I–II)	39	0.83	0.19–3.75	0.812	< 0.01	–
Malassezia-specific IgE class (III–VI vs. 0–II)	39	2.29	0.48–10.88	0.299	0.04	–

Notes: ECP, eosinophil cationic protein; ELR, Eosinophil-to-lymphocyte ratio; OR, odds ratio; 95% CI, 95% Wald confidence interval for the OR; LDH, Lactate dehydrogenase; Nagelkerke R^2, Nagelkerke pseudo-R-squared; p (H–L), p-value from the Hosmer–Lemeshow goodness-of-fit test; SIRI, systemic inflammatory response index.

Table S4g. Univariate logistic regression: baseline predictors of EASI < 7 (Week 40).

Predictor	n	OR	95% CI	p-value	Nagelkerke R^2	p (H–L)
White blood cell count (×10³/μL)	39	1.05	0.69–1.59	0.832	~~<~~ 0.002	0.372
Absolute lymphocyte count (×10³/μL)	39	2.33	0.59–9.15	0.225	0.06	0.275
Absolute neutrophil count (×10³/μL)	39	0.88	0.50–1.55	0.660	0.01	0.108
Systemic inflammatory response index (SIRI)	39	0.57	0.24–1.36	0.204	0.06	0.274
Absolute monocyte count (×10³/μL)	39	0.49	0.01–23.22	0.716	< 0.01	0.657
Absolute eosinophil count (×10³/μL)	39	2.01	0.18–22.70	0.574	0.01	0.458
Eosinophil-to-lymphocyte ratio (ELR)	39	1.18	0.03–45.38	0.929	< 0.01	0.383
Eosinophil (%)	39	1.04	0.86–1.26	0.710	0.01	0.410
Lactate dehydrogenase (LDH, U/L)	38	0.99	0.98–1.01	0.255	0.05	0.318
Eosinophil cationic protein (ECP, μg/L)	39	~~1.07~~ 1.01	0.95–1.06	0.828	< 0.01	0.225
Total serum IgE (kU/L)) *[sensitivity]*	39	1.00	0.999–1.00	0.293	0.04	0.066
Malassezia-specific IgE (kUA/L)	39	0.98	0.95–1.02	0.377	0.03	0.105
Total serum IgE class (III–IV vs. 0/I–II)	39	0.43	0.10–1.98	0.280	0.04	–
Malassezia-specific IgE class (III–VI vs. 0–II)	39	0.83	0.21–3.38	0.798	< 0.01	–

Notes: ECP, eosinophil cationic protein; ELR, Eosinophil-to-lymphocyte ratio; OR, odds ratio; 95% CI, 95% Wald confidence interval for the OR; LDH, Lactate dehydrogenase; Nagelkerke R^2, Nagelkerke pseudo-R-squared; p (H–L), p-value from the Hosmer–Lemeshow goodness-of-fit test; SIRI, systemic inflammatory response index

Multiplicity statement

Because multiple biomarker–outcome–time point combinations were examined, ROC analyses were considered exploratory and no multiplicity correction was applied.

Sample size rationale and missing data handling

No formal sample-size calculation was undertaken; a consecutive convenience cohort comprising all eligible patients was analysed, and precision is conveyed throughout by confidence intervals. Missing data were confined to one baseline LDH value and one participant lost to follow-up after week 16; analyses were complete-case per variable (n = 39 at week 40), and robustness was assessed by reassigning the withdrawn participant as a responder and as a non-responder for each binary week-40 endpoint and by median-imputing the missing LDH value.

Participant flow diagram

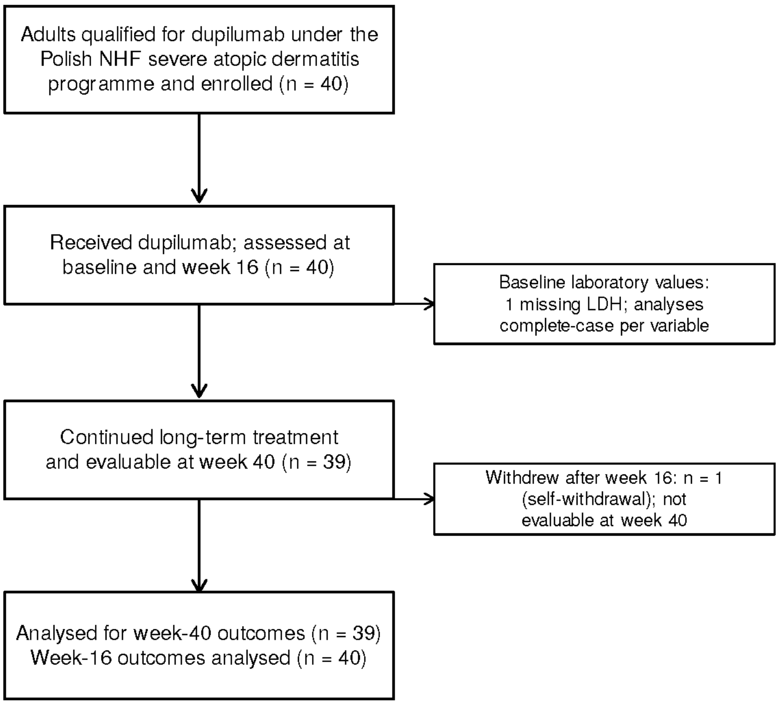

Figure S1. Participant flow. Forty patients received dupilumab and were assessed at baseline and week 16; one patient withdrew after the week-16 assessment and was not evaluable at week 40, yielding 39 participants analysed for week-40 outcomes. One baseline lactate dehydrogenase value was missing; laboratory analyses were performed complete-case per variable.
